# Defining Disease Phenotypes in Primary Care Electronic Health Records by a Machine Learning Approach: A Case Study in Identifying Rheumatoid Arthritis

**DOI:** 10.1371/journal.pone.0154515

**Published:** 2016-05-02

**Authors:** Shang-Ming Zhou, Fabiola Fernandez-Gutierrez, Jonathan Kennedy, Roxanne Cooksey, Mark Atkinson, Spiros Denaxas, Stefan Siebert, William G. Dixon, Terence W. O’Neill, Ernest Choy, Cathie Sudlow, UK Biobank Follow-up and Outcomes Group, Sinead Brophy

**Affiliations:** 1 Institute of Life Science, College of Medicine, Swansea University, Swansea, United Kingdom; 2 UCL Institute of Health Informatics and Farr Institute of Health Informatics Research, London, United Kingdom; 3 Institute of Infection, Immunity and Inflammation, University of Glasgow, Glasgow, United Kingdom; 4 Arthritis Research UK Centre for Epidemiology, Institute of Inflammation and Repair, Faculty of Medical and Human Sciences, Manchester Academic Health Science Centre, University of Manchester, Manchester, United Kingdom; 5 Arthritis Research UK CREATE Centre and Welsh Arthritis Research Network, School of Medicine, Cardiff University, Cardiff, United Kingdom; 6 Centre for Clinical Brain Sciences, University of Edinburgh, Edinburgh, United Kingdom; 7 The UK Biobank, Stockport, United Kingdom; University of Catania, ITALY

## Abstract

**Objectives:**

1) To use data-driven method to examine clinical codes (risk factors) of a medical condition in primary care electronic health records (EHRs) that can accurately predict a diagnosis of the condition in secondary care EHRs. 2) To develop and validate a disease phenotyping algorithm for rheumatoid arthritis using primary care EHRs.

**Methods:**

This study linked routine primary and secondary care EHRs in Wales, UK. A machine learning based scheme was used to identify patients with rheumatoid arthritis from primary care EHRs via the following steps: i) selection of variables by comparing relative frequencies of Read codes in the primary care dataset associated with disease case compared to non-disease control (disease/non-disease based on the secondary care diagnosis); ii) reduction of predictors/associated variables using a Random Forest method, iii) induction of decision rules from decision tree model. The proposed method was then extensively validated on an independent dataset, and compared for performance with two existing deterministic algorithms for RA which had been developed using expert clinical knowledge.

**Results:**

Primary care EHRs were available for 2,238,360 patients over the age of 16 and of these 20,667 were also linked in the secondary care rheumatology clinical system. In the linked dataset, 900 predictors (out of a total of 43,100 variables) in the primary care record were discovered more frequently in those with versus those without RA. These variables were reduced to 37 groups of related clinical codes, which were used to develop a decision tree model. The final algorithm identified 8 predictors related to diagnostic codes for RA, medication codes, such as those for disease modifying anti-rheumatic drugs, and absence of alternative diagnoses such as psoriatic arthritis. The proposed data-driven method performed as well as the expert clinical knowledge based methods.

**Conclusion:**

Data-driven scheme, such as ensemble machine learning methods, has the potential of identifying the most informative predictors in a cost-effective and rapid way to accurately and reliably classify rheumatoid arthritis or other complex medical conditions in primary care EHRs.

## Background

Rheumatoid arthritis (RA) is the most common chronic inflammatory arthritis worldwide. It affects approximately 1% of the population in countries like the UK [1] and USA [2], with significant morbidity and increased mortality. Most RA clinical trials capture relatively short-term data in small numbers of patients, while longer trials are costly and time-consuming, with difficulty maintaining the levels of follow up required to evaluate the important long-term outcomes in this chronic, life-long disease. Emerging studies have shown that patient recruitment for clinical trials and follow up from trials could be significantly improved by use of electronic health records [3][4][5][6]. But a key requirement is the accurate and reliable identification of patients who satisfy the RA criteria from electronic health records.

Indeed, in this age of big data, large quantities of real time data from national and regional population datasets can be accessed and utilised to gain knowledge on the entire population rather than extrapolating from a single sample. It is now possible to examine a patient’s journey through the healthcare system by linking their electronic health records [7,8]. One can examine information on first symptoms, tests/results, referrals, diagnosis, and prescriptions for all patients with a condition in the region or country, providing an unparalleled opportunity to study disease in a real world setting. This offers the chance for real time surveillance of disease history, co-morbidities and long term treatment effects in patients with chronic diseases, such as RA. However, since the introduction of biologic therapies, patients with RA are generally diagnosed and managed in specialist rheumatology centres, with relatively limited input from primary care physicians. As such, secondary care electronic health records contain more robust RA-related diagnostic data than primary care records, but these records are sparse, cover far smaller patient numbers, often contain only severe active disease and are not easily available. This leads to only identifying patients with RA in that specific rheumatology secondary care setting, thereby introducing bias and limiting generalizability. In contrast, primary care data is more widely available containing a much broader spectrum of patient care codes including details of a patient’s diagnoses, medications, managements and health outcomes and information on demographic variables and co-morbidies. But, in primary care EHRs, the non-specific diagnosis of ‘arthritis’ can be used for people who have other conditions like osteoarthritis, ankylosing spondylitis or non-specific joint pain [9] meaning that some conditions may appear under-reported in primary care while others over-reported. So accurately and reliably identifying patients with RA from primary care database remains challenging, and usually requires a combination of evidence from diagnosis, medication, treatment and investigation codes [10,11]. Existing algorithms to identify RA from primary care records are based on manual selection of the most relevant codes [12]. Thomas et al ‘s method [12] is based on codes chosen manually by clinical experts, while the other method [13] is based on the codes used by the Quality and Outcomes Framework (QOF), a pay for performance scheme in which a standardised set of clinician-selected codes is used to determine the prevalence of a condition (in this case RA) in a practice. However, the methodology of manual selection of relevant codes based on expert knowledge is very subjective and depends on the health care system of the area and what clinicians (often secondary care physicians) think should be found in the primary care record.

An alternative is to use secondary care diagnosis as a gold standard and examine which health-related codes in the primary care dataset are predictive of a secondary care diagnosis. That leads to patterns found in the primary care records can be used to predict the presence or absence of the RA in secondary care specialist data. This means RA can be reliably identified using primary care records in the absence of access to secondary care specialist records. But identifying these patterns is not straightforward, because the criteria which characterise the RA are buried within complex hierarchical terminology structures across multiple data points in the electronic health records of a patient, such as the Read codes (5 bytes, Version 3)[14] developed by the National Health Service of the United Kingdom (NHS), or the SNOMED CT (Systematized Nomenclature of Medicine Clinical Terms) that merges two large-scale terminologies—the Read codes and the SNOMED Reference terminologies developed by the College of American Pathologists [15]. In particular, recording practices with these terminologies vary substantially across healthcare settings depending on the purpose for which the data are being collected. As a result, a huge number of health-related variables (codes) often turn up to describe the conditions of a patient across multiple data points. This presents a big methodological challenge for identifying patients from electronic health records, as the high dimensionality of health-related features makes the classical statistical framework no longer feasible due to the curse of dimensionality [10,11][16][17]. Thus, to reliably distinguish RA patients from electronic health records, the key is to identify the most predictive RA code patterns buried in the electronic health records.

In this study, we aim to develop a robust, valid and cost-effective method of identifying the most informative predictors to detect RA patients from primary care electronic health records. Different from a purely knowledge driven and deterministic approach, in this study, we propose to use data-driven method, which were initially used in the computing and retail industries (e.g., looking at shopping patterns for marketing) [18], to identify patterns within the patient’s pathway of care to improve the accuracy of identification of those with RA from the primary care record. We link primary and secondary care electronic patient records, and identify patients with RA in the primary care dataset while using the secondary care diagnosis as the gold standard. Then we compare our data-driven methodology with two other existing expert knowledge based methods. If the data driven method performs as well as expert opinion, then it could provide a rapid and cost-effective way to reliably identify people with a health condition, especially where knowledge-driven algorithms do not already exist. Thus, the overall aim of this study was to develop an algorithm to define RA from primary care records using data driven method.

## Methods

### Dataset Preparations

The Farr Institute of Health Informatics Research comprises four centres distributed across the UK. One of the nodes, CIPHER (Centre for Improvement in Population Health through E-Records), brings together routine health data using the Secure Anonymised Information Linkage (SAIL) databank [19], which anonymously links a wide range of person-based data employing a unique personal identifier [20]. In this study the primary care general practice (GP) records were linked with the local rheumatology secondary care clinical system. The GP system uses Read codes, which are 5-digit codes that relate to diagnosis, medication and process of care.

The structure of Read codes allows almost anything to be coded in the patient EHRs, such as occupation; social circumstances; ethnicity and religion; clinical signs, symptoms and observations; laboratory tests and results; diagnoses; medications; a variety of administrative items. Read terms are organised by Chapters, such as Chapters 1 to 9 about history, examination, procedures and administration, Chapters A to U about conditions, diagnoses and injuries, and Chapters a to s about medications.

The secondary care dataset used in this study is from the clinical system—Cellma [21] which has been used in the rheumatology departments of local hospitals in Abertawe Bro Morgannwg University (ABMU—Swansea and Bridgend areas) and Cardiff and Vale University Health Board (CVUHB). This commercial system uses SNOMED-CT to code diagnosis and medications [22] as well as recording clinical data entered by rheumatologists at the point of capture. The Cellma systems were available in the ABMU region from March 2009 until October 2012, and in Cardiff from October 2013 to July 2014. Hospital admissions data for the whole population of Wales is available in the SAIL databank, along with data from all general practices in the ABMU region and 50% of general practices in the Cardiff region.

There were 2,238,360 patients over the age of 16 in the GP database contained in the SAIL databank between the years 1999–2013. Linking the general practice and Cellma databases gave an overlap of 20,667 people (15,459 from ABMU for 2009–2012 and 5,208 from Cardiff for 2013–2014). The proportion of patients with a diagnosis of RA, according to specialist rheumatologist consultants, recorded in Cellma (used as the gold standard diagnosis) was 17% (n = 2,588 overall, 2,029 in the ABMU region and 559 in the Cardiff region).

### Analysis Methods

#### Development of the data driven algorithm

Due to the higher number of patients with RA in ABMU, this area was used to develop the algorithm as outlined below. Then the Cardiff dataset was used as an independent population to validate the algorithm.

**Phase 1: Preliminary selection of predictors:** Due to the large number of codes available in the UK primary care system, a preliminary selection of codes associated with RA was needed. This involved examination of the relative frequencies of raw Read codes in those with gold standard Cellma diagnosis of RA (cases) versus all those without RA (controls), which is defined as follows

rFeq(x)=p(RA|x)−p(nonRA|x)

where *p*(*RA*|*x*) = *N*_*x*.1_/*N* and *p*(*nonRA*|*x*) = *N*_*x*.2_/*N* are the prior probabilities of the RA and non-RA cases respectively given the Read code *x* with *N*_*x*.*1*_ cases of RA and *N*_*x*.*2*_ cases of non-RA among all *N* patients. In ranking the Read codes, the defined relative frequency allows important but infrequent Read codes to be identified. For example, the code for ‘swollen hand’ may be rare in the entire database but 10 times more prevalent in RA patients. All the Read codes with a higher relative frequency in cases versus controls were selected as initial predictor variables. However, in this phase, the proposed relative frequency is a univariate metric only used to measure the significance of a single risk factor, without considering the interactions between different factors.

**Phase 2: Reduction of predictors through aggregation and wrapper approach:** In this phase, in order to select important subset of variables and taking into account the interactions among variables, a feature selection wrapper approach was employed to perform feature selection and classification tasks simultaneously. Random Forest was used to rank the importance of individual predictors and select key ones. Random Forest is an ensemble machine learning classifier consisting of many local classification and regression tree classifiers [23]. In our study, we used the Random Forest Gini impurity metric to rank the Read codes according to which were most useful in correctly classifying cases versus controls. The Gini quantity gives an indication of how strong the overall discriminative ability of a particular variable was for the classification problem under study. Importantly, the “grouping property” of sub-trees [24] enables the Random Forest to adeptly deal with correlation and interaction among variables. Emerging evidence has shown that ranked variables considering the correlation and interaction effects lead to a vast improvement for construction of a compact classification model [25]. The Random Forest method selects two thirds of the data to construct each of the trees and then employs the remaining one third of the data to estimate how many people are misclassified. For example, one tree might be: *If code for RA and code for methotrexate are present then identify these records as RA (case)*. Then the final one third split data can be used to see how often this tree correctly classifies a person. It performs this for all trees and then, generates a list of predictors ordered by their importance.

After ranking, an aggregation method was used to aggregate these selected Read codes if they were similar to others in the database. For example, some medication Read codes, such as those related to vitamin D prescription at a specific dose, were found among the list of selected predictors. Read codes for vitamin D at differing doses (perhaps less frequently prescribed but still valid predictors) can then be selected and examined to see if they improve the number of people correctly classified. This means that the list of initial predictors acts like a list of ‘seeds’ to identify similar variables in the database. The variables sharing the same condition can be further aggregated into one variable (e.g., grouping codes for laboratory tests that are normally performed together, such as Read codes for white blood cell count to include basophil count, lymphocyte count, eosinophil count, neutrophil count, and monocyte count). Although the Random Forest method constructed numerous possible trees that perform well in selecting important predictors to classify patients as either RA or non-RA, it is a “black-box” method in nature, as it is difficult to induce the transparent classification rules and interpret the predictions made by the model.

**Phase 3: Classification rule induction:** During this phase, the C5.0 decision tree (an improved version of C4.5) [26] was used to automatically create ‘*if-then*’ rules using the aggregated and selected predictors from the previous phases. Distinct from other machine learning techniques, decision trees integrate the functions of the classification and feature selection in one model structure. The C5.0 decision tree method uses a single tree/model to classify patients, while the ‘*if-then*’ statements generated by a tree define a unique route to one terminal node (RA or not RA) for any samples. For example, *if a diagnosis of PsA (Psoriatic Arthritis) was recorded*, *then the result would be ‘not RA’*. *If there was no diagnosis of PsA*, *then the algorithm would advance to the next section of the tree*. Each branch gives a single rule. This hierarchical structure leads to many rules sharing the same initial conditions, which constructs an algorithm of identifying RA.

#### Validation of the algorithm

The algorithm was then applied to two datasets: (a) the independent Cardiff dataset of patients with a GP and secondary care rheumatology record, to evaluate performance at classifying RA among those attending a rheumatology clinic; and (b) all 475,580 people in the GP system and resident within the ABMU area during the period March 2009 to October 2012 (this is the period when there are valid diagnosis of conditions within the ABMU Cellma) and aged 16+ during this time period. The performance of the algorithm in the Cardiff dataset was assessed by calculating the sensitivity, specificity and positive predictive value and compared with the performance of two existing algorithms which were generated based on expert clinical opinion. These two existing algorithms were (1) the Quality and Outcomes Framework (QOF) definition [13], and (2) the algorithm published by Thomas et al. [12], which was developed on data from GP systems in England and so comparable to data systems in Wales. (The codes used by the QOF criteria and Thomas et al’s method in this study can be found in the S1 Table). In terms of dataset (b) we reported sensitivity, specificity and positive predictive value using a best case/worst case scenario analysis (Tables 1, 2 and 3). We also examined the prevalence of RA over time using these algorithms in dataset (b) to confirm that estimates were within the range expected from the literature.

**Table 1:** The performance of data mining algorithm given worst case/best case assumptions.

		Outpatient data (Cellma- ABMU)		
		RA	Not RA	No data
GP data	**RA**	1323	396	2560
	**Not RA**	265	10084	459727
	**No data**	138	1087	
	Worst case*: Sensitivity 80%, Specificity 99%, Positive predictive value 30%		Best case**: Sensitivity 93%, Specificity 99%, Positive predictive value 90.7%	

*Worst case: no record in outpatients signifies not RA.

**Best case: no record in outpatients signifies patient with RA treated elsewhere.

**Table 2:** The performance of QOF criteria given worst case/best case assumptions.

		Outpatient data(Cellma- ABMU)		
		RA	Not RA	No data
GP data	**RA**	1377	513	2851
	**Not RA**	211	9967	459436
	**No data**	138	1087	
	Worst case: Sensitivity 86.7%, Specificity 99%, Positive predictive value 29%		Best case: Sensitivity 95%, Specificity 99%, Positive predictive value 89%	

**Table 3:** The performance of expert knowledge driven algorithm given worst case/best case assumptions.

		Outpatient data(Cellma- ABMU)		
		RA	Not RA	No data
GP data	**RA**	1333	570	2139
	**Not RA**	255	9910	460148
	**No data**	138	1087	
	Worst case: Sensitivity 83.9%, Specificity 99%, Positive predictive value 28%		Best case: Sensitivity 94.2%, Specificity 99%, Positive predictive value 88%	

#### Statistical analysis

The algorithm implementation, parameter tuning and performance evaluation were all performed using R language in RStudio version 3.0.2 [27] with the packages of ‘*randomForest’* for RF [28], and *‘C50’* for C5.0 trees [29]. The performance of the algorithm was assessed in terms of the sensitivity, specificity and positive predictive value.

## Results

### Algorithm Development

**Fig 1:**
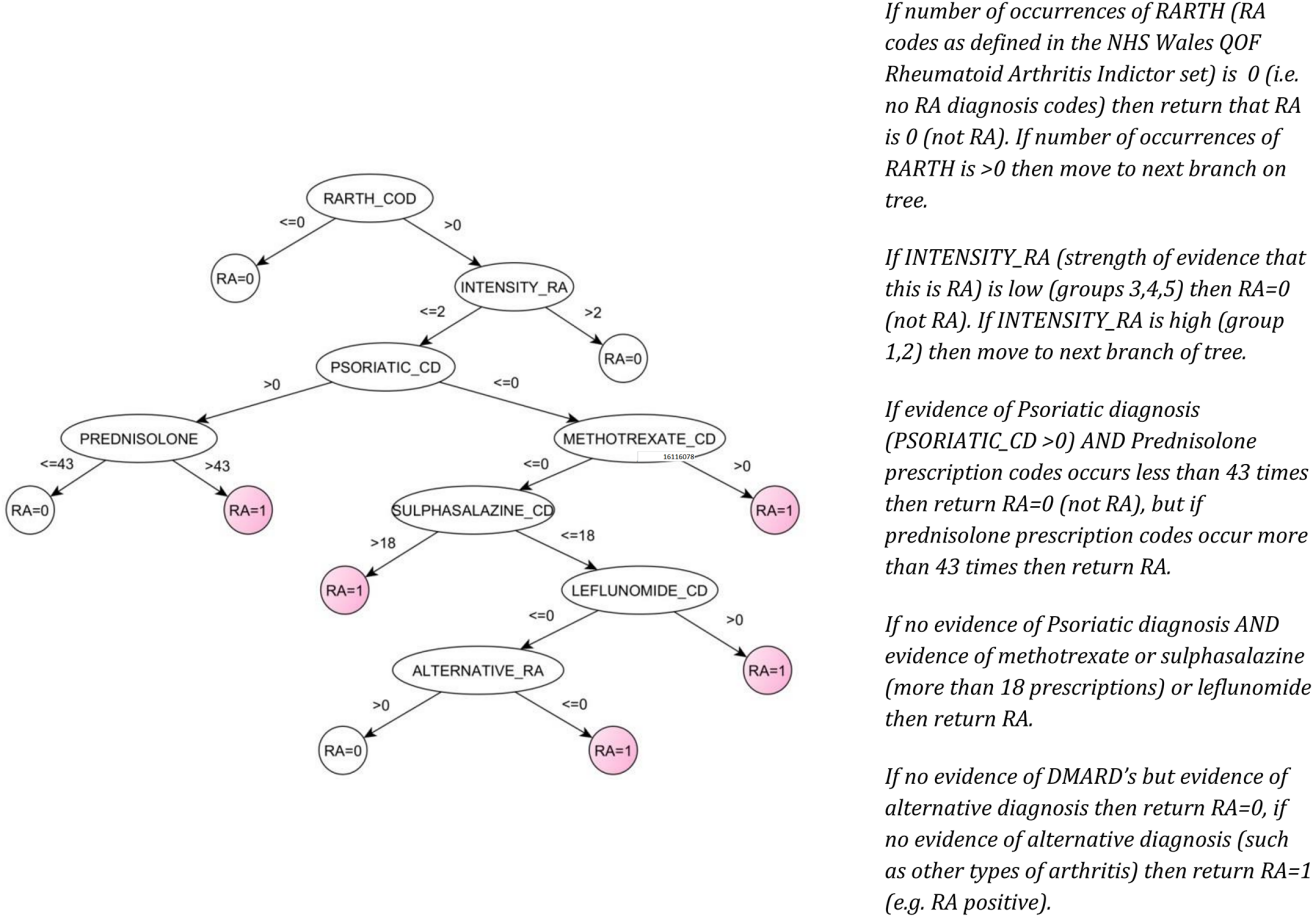
RA phenotyping model: (Left) C5.0 decision tree. The value of a variable is the number of its occurrence. At each final node, RA = 1 indicates classification for positive RA and RA = 0 negative classified for RA. (Right) Decision rules generated from the C5.0 decision tree.

1. Phase 1: 900 predictor codes were identified from 43,100 potential Read codes. These included different codes identifying, for example, the same medication but at varying doses or varying administration methods (tablet, syrup, injection). These similar codes were aggregated in the next phase.
2. Phase 2: The 900 predictors were reduced to 37 predictor code groups after the aggregation phase. The 37 predictor code groups included groups of codes under a single name (for example the code of ‘PREDNISOLONE’ would include all codes with Prednisolone listed as the active ingredient, including the Read code for PREDNISOLONE 2.5 mg e/c tablets and the code for PREDNISOLONE 5mg e/c tablets).
3. Phase 3: A C5.0 decision tree was generated to identify RA patients. The C5.0 tree further reduced the 37 predictor code groups to 8 predictor code groups. The decision tree method helps to remove codes which cluster with other more predictive codes, accounting for covariance and allowing the simplest model to be identified with the highest positive predictive value in the Welsh population (see Fig 1). The 8 predictor code groups for the Welsh population were: (1) RA codes as defined in the NHS QOF indictor set, (2) a group of codes indicating strength of evidence for RA (including codes for seropositive or erosive RA and codes for systemic manifestations of RA, see Table 4 for details), (3) a group of psoriatic arthritis Read codes used to exclude RA; (4) an additional group of alternative diagnosis codes for arthropathies other than RA or PsA also used to exclude RA as a diagnosis, (5) prednisolone (at all different dosage), (6) methotrexate (all Disease-Modifying Antirheumatic Drugs (DMARD) with the active ingredient listed as methotrexate), (7) sulphasalazine and (8) leflunomide.

**Table 4:** Algorithm code definitions.

*Predictor*	*Description*
RARTH_COD	Rheumatoid Arthritis codes as defined in the NHS Wales QOF Rheumatoid Arthritis Indicator Set (RA Indicator Set NHS QOF Wales, 2014)
INTENSITY_RA	A categorical variable with 5 levels, inspired in the reasoning on Thomas et al (2008) work to define intensity of rheumatoid conditions: Class 1 is the most severe diagnosis of rheumatoid arthritis and includes sero-positive rheumatoid arthritis. Class 2 contains codes related to classification of rheumatoid arthritis. Class 3 contains conditions that are related to rheumatoid arthritis, such as rheumatoid nodule. Class 4 identifies patients that have sero-negetive rheumatoid arthritis. The final class is class 9 and identifies patients who do not have any history of rheumatoid arthritis.
ALTERNATIVE_RA	Alternative arthropathy Read codes inspired in the reasoning in Thomas et al (2008).
PSORIATIC_CD	Psoriatic Arthritis Read codes
PREDNISOLONE	Read codes related to different dosage of Prednisolone. Prednisolone is an corticosteroid drug for controlling a local flare in a joint, which is used effectively in combination with other drugs (Ter Wee et al., 2014).
METHOTREXATE_CD	Disease-modifying Antirheumatic Drugs (DMARDs) grouped by their active ingredient- methotrexate,
SULPHASALAZINE_CD	DMARDs grouped by their active ingredient-sulphasalazine
LEFLUNOMIDE_CD	DMARDs grouped by their active ingredient-leflunomide

The decision rules with these predictor codes were induced as shown in Fig 1.

### Algorithm Validation

#### Secondary care/GP overlap population in Cardiff

The population used for the validation (Cardiff-Cellma) had 27% prevalence of RA. The data driven algorithm gave a PPV: 85.6%, specificity: 94.6%, sensitivity: 86.2% and overall accuracy of 92.29%. This compared with the QOF criteria alone (which is included within the data driven algorithm) which gave PPV: 83%, specificity: 93.2%, sensitivity: 89.1% and accuracy: 92%. The Thomas et al’s method gave PPV: 78.5%, specificity: 91.1%, sensitivity: 86.2% and accuracy: 89.8%.

#### Primary care population

**Fig 2:**
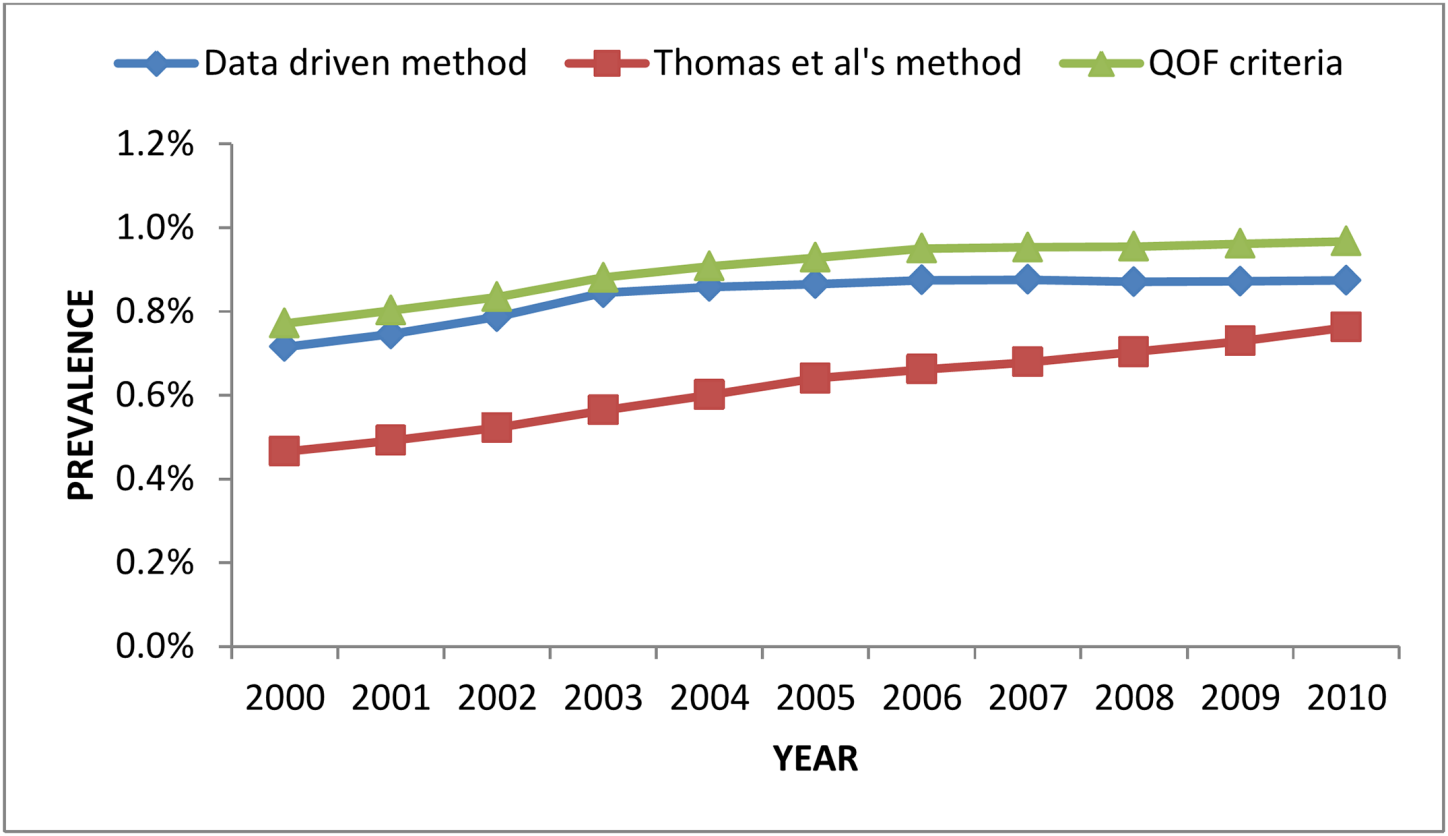
Prevalence of RA for 2000–2010 in the ABMU region, Wales, UK.

Of the 475,580 people over 16 years old in the GP system in the ABMU area, the algorithm would have classified 4,279 (0.9%) as having RA, which is consistent with an existing study of estimated prevalence of RA across the UK population (0.8–0.9%) [1]. Of the 4,279 people, 1,719 (40%) were found in the secondary care rheumatology system and 1,323 (77%) were confirmed as having RA by the rheumatologist. However, 2,560 (60%) of all the RA cases in the GP data were not found in the secondary care system. This could be because (a) they did not have RA, (b) they are treated outside the ABMU area, such as elsewhere in Wales or in England, (c) they are new cases waiting for a referral to the rheumatologist, or (d) they are old cases and no longer regularly seeing a rheumatologist or did not see a rheumatologist in the time period for which we have data. If we assume a worst case scenario, that none of the 2,560 cases have RA then this would give a prevalence of RA of 0.33%, a PPV: 30.9%, specificity: 99%, sensitivity 83%. If we assume a best case scenario, that all of the 2,560 cases did have RA but were treated by a rheumatologist outside the area then this estimate would give a RA prevalence of 0.93%, with PPV: 91.3%, specificity: 99.9%, and sensitivity 94%. Plotting the prevalence of RA within the ABMU region over time (Fig 2) shows that the data-driven model and QOF algorithm produced comparable results, while the Thomas et al criteria gave a lower prevalence for the region.

## Discussion

EHRs have been considered as an essential component in delivering high quality healthcare. They provide enormous benefits not only for patient care, but also, when linked with other sources of health-related records, for public health surveillance, measurement of outcomes and a wide variety of research studies [16][17][30][31]. However, it is recognised that there are large variations in the accuracy and completeness of the clinical information stored in primary care electronic patient records [32][33]. Issues in using primary care electronic patient data include: the codes are not robust enough; data curation was not performed resulting in a disconnection between data elements in the record and the patient's clinical status; and clinicians may fail to enter codes properly, particularly where these relate to conditions diagnosed predominantly in secondary care settings. Therefore, it is unclear whether current primary care electronic patient records capture clinical data adequately to reliably identify patients with a particular condition for research purposes. In this study, we link primary care records with secondary care records, using the secondary care data acting as gold standard diagnosis for RA, to develop a rapid data-driven method which can be used to accurately identify patients with RA in primary care data codes. The developed algorithm performed as well as expert opinion and the used QOF criteria in the UK. It demonstrates a method of deriving algorithms which can be used for conditions without having QOF criteria and could be extended to develop algorithms on systems that do not use Read codes. This data-driven method is based on the RA patient pathway found in the health care system so can be used to derive algorithms specific for that system. The findings demonstrate that for RA the QOF is a reliable and easy way of identifying a condition. Therefore, when deriving algorithms, looking at the QOF criteria may be the best place to start in the UK NHS. This finding may also be true in other countries where coding is linked to financial payments as there may be less variance around these codes [34]. However, the UK QOF criteria only apply to a limited number of conditions, while many countries do not have robust coding linked to financial payment. In these cases, the data driven method described here is a reliable way to identify codes related to RA.

The algorithm derived here relies on medication codes which improves the positive predictive value, but reduces sensitivity and importantly will mainly select patients with a longer history of the RA condition (who will have had time to be given different types of medication) and more severe disease (requiring more therapies). Previous studies have also reported that adding codes for *DMARDs* from administration ICD billing data increases positive predictive value in classifying RA patients, but decreases sensitivity [35][36]. Therefore, medication codes can feature highly in a prevalence algorithm but an incidence algorithm may need to focus more strongly on tests/procedures and ensuring no alternative diagnosis. Thus, distinct algorithms to define prevalent and incident cases may be required.

This study offers several advantages. 1) It provides a novel way of automatically selecting the most informative variables for RA diagnosis from a large number of coded data elements. This can bring benefits in health informatics, for example, reducing the costs from many aspects, such as model construction, data usages; using only the most informative variables can also simplify the problem and speed up solutions; by selecting only the most informative variables, it is feasible to employ a particular classification model due to the challenge of curse of dimensionality; in addition, discarding the irrelevant features can clean data and remove the noises to significantly improve the generalisation performance by avoiding over-fitting dilemma. 2) It can be performed very rapidly and cost-effectively and is particularly useful in situations where it is unclear what RA codes should be used in the case of heterogeneous or complex conditions. 3) The proposed method uses real life patient pathways to classify RA rather than idealised best practice. 4) The RA decision rules generated are easily understood by users and clinicians in practical applications. It is a transparent method, which gives objective results and is repeatable, so that all data and the method constructed can themselves lead to reproducible research (datasets and methods can be published to be independently analysed).

However, the method we used requires a gold standard in order to develop or train the model. There has to be a ‘correctly diagnosed’ reference in order to detect the patterns that predict that reference. In health informatics research, a key limitation in any algorithm development research is the lack of a population based gold standard and without this, obtaining a good estimate of performance of models applied to the whole population is difficult. This is illustrated by the broad range of possible performance statistics for our RA algorithm when worst and best case scenarios are considered. In addition, this data-driven method is mainly applicable when using coded clinical terms and may not be applicable to textual data from notes and letters, as these will require prior interpretation in order to be able to detect patterns.

Regarding implications for future research and practice, while manual selection based methods may achieve good predictive performance in patient phenotype identification for specific disease, they require significant interactions between domain experts and informaticians to create algorithms for each disease, which significantly limits their ability to be expanded to different disease phenotypes. The data-driven approach in this study aimed at identifying the most informative clinical criteria from data independently of domain experts (and the subjectivity associated with this) and thus offers great promise of generalisation and scalability to phenotype identification for RA and other rheumatic diseases.

The findings of this work demonstrate how machine learning methods can be utilized to create reliable disease phenotypes in EHRs. This method may be particularly valuable for large population-based research cohorts like UK Biobank [37][38], USA eMERGE network [39][40][41], to give simple algorithms with good performance that are transparent and easy to apply. This paper has also compared the data-driven methods with the two existing RA algorithms available for RA research using UK datasets, so offers a comparison of performance that can be used by researchers to decide which algorithm is most appropriate to their research.

## Supporting Information

S1 Table Aggregated Read Codes Used in QoF Criteria, Thomas et al’s Model and Data-Driven Model. (DOCX)
