# Supplementary material for: Defining Disease Phenotypes in Primary Care Electronic Health Records by a Machine Learning Approach: A Case Study in Identifying Rheumatoid Arthritis
Source: PLoS One. 2016 May 2;11(5):e0154515. doi: 10.1371/journal.pone.0154515 (PMC4852928; doi:10.1371/journal.pone.0154515)
Supplement: S1 Table — (DOCX) [file pone.0154515.s001.docx]

**Aggregated Read Codes Used in QoF Criteria, Thomas et al’s Model and Data Driven Model**

| NHS QOF Rheumatoid Arthritis Indicators | |
| --- | --- |
| **Group:** | RARTH_COD |
| **Description:** | NHS QOF Rheumatoid Arthritis indicator |
|  |  |
| **READ CD** | **Description** |
| N040. | Rheumatoid Arthritis |
| N0400 | Rheumatoid arthritis of cervical spine |
| N040T | Flare of rheumatoid arthritis |
| N0402 | Rheumatoid arthritis of shoulder |
| N040S | Rheumatoid arthritis - multiple joint |
| N0409 | Rheumatoid arthritis of PIP joint of finger |
| N0408 | Rheumatoid arthritis of MCP joint |
| N0401 | Other rheumatoid arthritis of spine |
| N0400 | Rheumatoid arthritis of cervical spine |
| N0407 | Rheumatoid arthritis of wrist |
| N040B | Rheumatoid arthritis of hip |
| N040D | Rheumatoid arthritis of knee |
| N040K | Rheumatoid arthritis of 1st MTP joint |
| N040F | Rheumatoid arthritis of ankle |
| N0405 | Rheumatoid arthritis of elbow |
| N040A | Rheumatoid arthritis of DIP joint of finger |
| N0406 | Rheumatoid arthritis of distal radio-ulnar joint |
| N040H | Rheumatoid arthritis of talonavicular joint |
| N040J | Rheumatoid arthritis of other tarsal joint |
| N040G | Rheumatoid arthritis of subtalar joint |
| N040L | Rheumatoid arthritis of lesser MTP joint |
| N040C | Rheumatoid arthritis of sacro-iliac joint |
| N0404 | Rheumatoid arthritis of acromioclavicular joint |
| N040N | Rheumatoid vasculitis |
| N040R | Rheumatoid nodule |
| N040P | Seronegative rheumatoid arthritis |
| N041. | Felty's syndrome |
| N042. | Other rheumatoid arthropathy with visceral or systemic involvement (Excluding N0420 Rheumatic carditis (v27)) |
| N0422 | Rheumatoid nodule |
| N042z | Rheumatoid arthropathy + visceral/systemic involvement NOS |
| N0421 | Rheumatoid lung disease |
| N047. | Seropositive errosive rheumatoid arthritis |
| N04X. | [X]Seropositive rheumatoid arthritis, unspecified |
| N04y0 | Rheumatoid lung |
| N04y2 | Adult onset Still's disease |
| Nyu11 | [X]Other seropositive rheumatoid arthritis |
| Nyu12 | [X]Other specified rheumatoid arthritis |
| Nyu1G | [X]Seropositive rheumatoid arthritis, unspecified |
| Nyu10 | [X]Rheumatoid arthritis+involvement/other organs or systems |
| G5yA. | Rheumatoid carditis |
| G5y8. | Rheumatoid myocarditis |

| Data Driven Model | |
| --- | --- |
| **Group:** | RARTH_COD |
| **Description:** | NHS QOF Rheumatoid Arthritis indicator |
|  |  |
| **READ CD** | **Description** |
| N040. | Rheumatoid Arthritis |
| N0400 | Rheumatoid arthritis of cervical spine |
| N040T | Flare of rheumatoid arthritis |
| N0402 | Rheumatoid arthritis of shoulder |
| N040S | Rheumatoid arthritis - multiple joint |
| N0409 | Rheumatoid arthritis of PIP joint of finger |
| N0408 | Rheumatoid arthritis of MCP joint |
| N0401 | Other rheumatoid arthritis of spine |
| N0400 | Rheumatoid arthritis of cervical spine |
| N0407 | Rheumatoid arthritis of wrist |
| N040B | Rheumatoid arthritis of hip |
| N040D | Rheumatoid arthritis of knee |
| N040K | Rheumatoid arthritis of 1st MTP joint |
| N040F | Rheumatoid arthritis of ankle |
| N0405 | Rheumatoid arthritis of elbow |
| N040A | Rheumatoid arthritis of DIP joint of finger |
| N0406 | Rheumatoid arthritis of distal radio-ulnar joint |
| N040H | Rheumatoid arthritis of talonavicular joint |
| N040J | Rheumatoid arthritis of other tarsal joint |
| N040G | Rheumatoid arthritis of subtalar joint |
| N040L | Rheumatoid arthritis of lesser MTP joint |
| N040C | Rheumatoid arthritis of sacro-iliac joint |
| N0404 | Rheumatoid arthritis of acromioclavicular joint |
| N040N | Rheumatoid vasculitis |
| N040R | Rheumatoid nodule |
| N040P | Seronegative rheumatoid arthritis |
| N041. | Felty's syndrome |
| N042. | Other rheumatoid arthropathy with visceral or systemic involvement (Excluding N0420 Rheumatic carditis (v27)) |
| N0422 | Rheumatoid nodule |
| N042z | Rheumatoid arthropathy + visceral/systemic involvement NOS |
| N0421 | Rheumatoid lung disease |
| N047. | Seropositive errosive rheumatoid arthritis |
| N04X. | [X]Seropositive rheumatoid arthritis, unspecified |
| N04y0 | Rheumatoid lung |
| N04y2 | Adult onset Still's disease |
| Nyu11 | [X]Other seropositive rheumatoid arthritis |
| Nyu12 | [X]Other specified rheumatoid arthritis |
| Nyu1G | [X]Seropositive rheumatoid arthritis, unspecified |
| Nyu10 | [X]Rheumatoid arthritis+involvement/other organs or systems |
| G5yA. | Rheumatoid carditis |
| G5y8. | Rheumatoid myocarditis |
|  |  |
|  |  |
| **Group:** | CVDASSRA_COD |
| **Description:** | CVD risk assessment tool adjusted for RA codes |
|  |  |
| **READ CD** | **Description** |
| 38DP. | QRISK2 cardiovascular disease 10 year risk score |
|  |  |
|  |  |
| **Group:** | CVDASSRAEXC_COD |
| **Description:** | CVD risk assessment tool adjusted for RA exception codes |
|  |  |
| **READ CD** | **Description** |
| 8IEV. | QRISK2 cardiovascular disease risk assessment declined |
| 9NSB. | Unsuitable for QRISK2 cardiovascular disease risk assessment |
|  |  |
|  |  |
| **Group:** | DXA2_COD |
| **Description:** | A DXA scan result with a T score value |
|  |  |
| **READ CD** | **Description** |
| 58EE. | Hip DXA scan T score |
| 58EK. | Lumbar spine DXA scan T score |
| 58ES. | Femoral neck DEXA scan T score |
|  |  |
|  |  |
| **Group:** | STRT_COD |
| **Description:** | Stroke or TIA codes |
|  |  |
| **READ CD** | **Description** |
| G61.. | Intracerebral haemorrhage |
| G610 | Cortical haemorrhage |
| G611. | Internal capsule haemorrhage |
| G612. | Basal nucleus haemorrhage |
| G613. | Cerebellar haemorrhage |
| G614. | Pontine haemorrhage |
| G615. | Bulbar haemorrhage |
| G616. | External capsule haemorrhage |
| G618. | Intracerebral haemorrhage, multiple localized |
| G61X. | Intracerebral haemorrhage in hemisphere, unspecified |
| G61X0 | Left sided intracerebral haemorrhage, unspecified |
| G61X1 | Right sided intracerebral haemorrhage, unspecified |
| G61z. | Intracerebral haemorrhage NOS |
| G63y0 | Cerebral infarct due to thrombosis of precerebral arteries |
| G63y1 | Cerebral infarction due to embolism of precerebral arteries |
| G64.. | Cerebral arterial occlusion |
| G640. | Cerebral thrombosis |
| G6400 | Cerebral infarction due to thrombosis of cerebral arteries |
| G641. | Cerebral embolism |
| G6410 | Cerebral infarction due to embolism of cerebral arteries |
| G64z. | Cerebral infarction NOS |
| G64z0 | Brainstem infarction |
| G64z1 | Wallenberg syndrome |
| G64z2 | Left sided cerebral infarction |
| G64z3 | Right sided cerebral infarction |
| G64z4 | Infarction of basal ganglia |
| G66.. | Stroke and cerebrovascular accident unspecified |
| G660. | Middle cerebral artery syndrome |
| G661. | Anterior cerebral artery syndrome |
| G662. | Posterior cerebral artery syndrome |
| G663. | Brain stem stroke syndrome |
| G664. | Cerebellar stroke syndrome |
| G665. | Pure motor lacunar syndrome |
| G666. | Pure sensory lacunar syndrome |
| G667. | Left sided CVA |
| G668. | Right sided CVA |
| G6760 | Cerebral infarct due cerebral venous thrombosis, nonpyogenic |
| G6W.. | Cerebral infarct due unspecified occlusion/stenosis precerebral arteries |
| G6X.. | Cerebral infarction due/unspecified occlusion or stenosis/cerebral arteries |
| G65.. | Transient cerebral ischaemia |
| G650. | Basilar artery syndrome |
| G651. | Vertebral artery syndrome |
| G6510 | Vertebro-basilar artery syndrome |
| G652. | Subclavian steal syndrome |
| G653. | Carotid artery syndrome hemispheric |
| G654. | Multiple and bilateral precerebral artery syndromes |
| G656. | Vertebrobasilar insufficiency |
| G657. | Carotid territory transient ischaemic attack |
| G65y. | Other transient cerebral ischaemia |
| G65zz | Transient cerebral ischaemia NOS |
| Gyu62 | [X]Other intracerebral haemorrhage |
| Gyu63 | [X]Cerebral infarction due/unspecified occlusion or stenosis/cerebral arteries |
| Gyu64 | [X]Other cerebral infarction |
| Gyu65 | [X]Occlusion and stenosis of other precerebral arteries |
| Gyu66 | [X]Occlusion and stenosis of other cerebral arteries |
| Gyu6F | [X]Intracerebral haemorrhage in hemisphere, unspecified |
| Gyu6G | [X]Cerebral infarct due unspecified occlusion/stenosis precerebral arteries |
| ZV12D | [V]Personal history of transient ischaemic attack |
| Fyu55 | [X]Other transient cerebral ischaemic attacks+related syndromes |
| G619. | Lobar cerebral haemorrhage |

|  |  |
| --- | --- |
| **Group:** | RARTHEXC_COD |
| **Description:** | Rheumatoid arthritis exception reporting codes |
|  |  |
| **READ CD** | **Description** |
| 9hR.. | Exception reporting: rheumatoid arthritis quality indicators |
| 9hR0. | Excepted from rheumatoid arthritis quality indicators: patient unsuitable |
| 9hR1. | Excepted from rheumatoid arthritis quality indicators: informed dissent |
|  |  |
|  |  |
| **Group:** | FRCASSRA_COD |
| **Description:** | Fracture risk assessment tool adjusted for RA codes |
|  |  |
| **READ CD** | **Description** |
| 38DB. | World Health Organisation FRAX 10 year hip fracture probability score |
| 38DC. | World Health Organisation FRAX 10 year osteoporotic fracture probability score |
| 38GR. | QFracture risk calculator |
| 38DB0 | World Health Organisation FRAX 10 year hip fracture probability score with bone mineral density |
| 38DB1 | World Health Organisation FRAX 10 year hip fracture probability score with body mass index |
| 38DC0 | World Health Organisation FRAX 10 year osteoporotic fracture probability score with bone mineral density |
| 38DC1 | World Health Organisation FRAX 10 year osteoporotic fracture probability score with body mass index |
|  |  |
|  |  |
| **Group:** | FRCASSRAEXC_COD |
| **Description:** | Fracture risk assessment tool adjusted for RA exception codes |
|  |  |
| **READ CD** | **Description** |
| 9OdB. | Osteoporosis risk assessment refused |
| 9OdC. | Osteoporosis risk assessment defaulted |
|  |  |
|  |  |
| **Group:** | DXA_COD |
| **Description:** | A DXA scan result of osteoporotic without a value |
|  |  |
| **READ CD** | **Description** |
| 58EG. | Hip DXA scan result osteoporotic |
| 58EM. | Lumbar DXA scan result osteoporotic |
| 58EV. | Femoral neck DEXA scan result osteoporotic |
|  |  |
|  |  |
|  |  |
|  |  |
|  |  |
|  |  |
| **Group:** | OSTEO_COD |
| **Description:** | Osteoporosis diagnostic codes |
|  |  |
| **READ CD** | **Description** |
| N330. | Osteoporosis |
| N3300 | Osteoporosis; unspecified |
| N3301 | Senile osteoporosis |
| N3302 | Postmenopausal osteoporosis |
| N3303 | Idiopathic generalised osteoporosis |
| N3304 | Dissuse osteoporosis |
| N3305 | Drug-induced osteoporosis |
| N3306 | Postoophorectomy osteoporosis |
| N3307 | Postsurgical malabsorption osteoporosis |
| N330A | Osteoporosis in endocrine disorders |
| N330B | Vertebral osteoporosis |
| N330C | Osteoporosis localized to spine |
| N330D | Osteoporosis due to corticosteroids |
| N330z | Osteoporosis NOS |
| N3312 | Postoophorectomy osteoporosis with pathological fracture |
| N3313 | Osteoporosis of disuse with pathological fracture |
| N3316 | Idiopathic osteoporosis with pathological fracture |
| N3318 | Osteoporosis + pathological fracture lumbar vertebrae |
| N3319 | Osteoporosis + pathological fracture thoracic vertebrae |
| N331A | Osteoporosis + pathological fracture cervical vertebrae |
| N331B | Postmenopausal osteoporosis with pathological fracture |
| N331H | Collapse of cervical vertebra due to osteoporosis |
| N331J | Collapse of lumbar vertebra due to osteoporosis |
| N331K | Collapse of thoracic vertebra due to osteoporosis |
| N331L | Collapse of vertebra due to osteoporosis NOS |
| N331M | Fragility fracture due to unspecified osteoporosis |
| NyuB0 | [X]Other osteoporosis with pathological fracture |
| NyuB1 | [X]Other osteoporosis |
| NyuB8 | [X]Unspecified osteoporosis with pathological fracture |
| N3314 | Postsurgical malabsorption osteoporosis with path fracture |
| N3315 | Drug-induced osteoporosis with pathological fracture |
| N3746 | Osteoporotic kyphosis |
| NyuB2 | [X]Osteoporosis in other disorders classified elsewhere |

| **Group:** | INTENSITY_RA |  |
| --- | --- | --- |
| **Description:** | RA intensity coded |  |
|  |  |  |
| **READ CD** | **Description** | **value** |
| ***** | No RA code | 9 |
| N047. | Seropositive errosive rheumatoid arthritis | 1 |
| N04X. | Seropositive rheumatoid arthritis, unspecified | 1 |
| Nyu1G | [X]Seropositive rheumatoid arthritis, unspecified | 1 |
| N040. | Rheumatoid arthritis | 2 |
| 14G1. | H/O: rheumatoid arthritis | 2 |
| N040T | Flare of rheumatoid arthritis | 2 |
| 66H.. | Rheumatoid arthrit. monitoring | 2 |
| N0402 | Rheumatoid arthritis of shoulder | 2 |
| N005. | Adult Still's Disease | 2 |
| N040S | Rheumatoid arthritis - multiple joint | 2 |
| N04y2 | Adult-onset Still's disease | 2 |
| N0409 | Rheumatoid arthritis of PIP joint of finger | 2 |
| N0408 | Rheumatoid arthritis of MCP joint | 2 |
| N0401 | Other rheumatoid arthritis of spine | 2 |
| N0400 | Rheumatoid arthritis of cervical spine | 2 |
| N0407 | Rheumatoid arthritis of wrist | 2 |
| N040B | Rheumatoid arthritis of hip | 2 |
| N040D | Rheumatoid arthritis of knee | 2 |
| N040K | Rheumatoid arthritis of 1st MTP joint | 2 |
| N040F | Rheumatoid arthritis of ankle | 2 |
| N0405 | Rheumatoid arthritis of elbow | 2 |
| N040A | Rheumatoid arthritis of DIP joint of finger | 2 |
| N0406 | Rheumatoid arthritis of distal radio-ulnar joint | 2 |
| Nyu12 | [X]Other specified rheumatoid arthritis | 2 |
| N040H | Rheumatoid arthritis of talonavicular joint | 2 |
| N040J | Rheumatoid arthritis of other tarsal joint | 2 |
| N040G | Rheumatoid arthritis of subtalar joint | 2 |
| Nyu11 | [X]Other seropositive rheumatoid arthritis | 2 |
| N040L | Rheumatoid arthritis of lesser MTP joint | 2 |
| 38DZ. | Disease activity score in rheumatoid arthritis | 2 |
| N040C | Rheumatoid arthritis of sacro-iliac joint | 2 |
| N0404 | Rheumatoid arthritis of acromioclavicular joint | 2 |
| N0422 | Rheumatoid nodule | 3 |
| H570. | Rheumatoid lung | 3 |
| N041. | Felty's syndrome | 3 |
| N040N | Rheumatoid vasculitis | 3 |
| N04y0 | Rheumatoid lung | 3 |
| N042z | Rheumatoid arthropathy + visceral/systemic involvement NOS | 3 |
| G5yA. | Rheumatoid carditis | 3 |
| N0421 | Rheumatoid lung disease | 3 |
| N042. | Other rheumatoid arthropathy + visceral/systemic involvement | 3 |
| G5y8. | Rheumatoid myocarditis | 3 |
| N040R | Rheumatoid nodule | 3 |
| F3712 | Polyneuropathy in rheumatoid arthritis | 3 |
| N040P | Seronegative rheumatoid arthritis | 4 |
| N04.. | Rheumatoid arthritis and other inflammatory polyarthropathy | 4 |
| 7P203 | Delivery of rehabilitation for rheumatoid arthritis | 4 |

| **Group:** | ALTERNATIVE_CD |
| --- | --- |
| **Description:** | Alternative Arthropathy codes |
|  |  |
| **READ CD** | **Description** |
| M160. | Psoriatic arthropathy |
| C34.. | Gout |
| N100. | Ankylosing spondylitis |
| N02.. | Crystal arthropathies |
| N023. | Gouty arthritis |
| N01w. | Reactive arthropathy, unspecified |
| N013. | Postdysenteric reactive arthropathy |
| A993. | Reiter's disease / syndrome |
| C340. | Gouty arthropathy |
| C342. | Idiopathic gout |
| M160z | Psoriatic arthropathy NOS |
| N023z | Gouty arthritis NOS |
| N015. | Arthropathy associated with other viral diseases |
| A56x0 | Arthritis due to rubella |
| E245. | Hallucinogen dependence |
| A9850 | Gonococcal arthritis |
| C34z. | Gout NOS |
| A15.. | Tuberculous of bones and joints |
| 6693. | Joints gout affected |
| A0223 | Salmonella arthritis |
| N011x | Sexually acquired reactive arthropathy of multiple sites |
| M1601 | Distal interphalangeal psoriatic arthropathy |
| N01w9 | Reactive arthropathy of hip |
| N10.. | Inflammatory spondylopathies |
| N0237 | Gouty arthritis of the ankle and foot |
| N014. | Arthropathy associated with other bacterial diseases |
| N015z | Arthropathy associated with other viral disease NOS |
| N0213 | Chondrocalcinosis-pyrophosphate crystals, of the forearm |
| N10yz | Other inflammatory spondylopathies NOS |
| N02zz | Crystal arthropathy NOS |
| N010A | Arthritis in Lyme disease |
| N10y. | Other inflammatory spondylopathies |
| C344. | Drug-induced gout |
| N02zD | Crystal arthropathy NOS, of wrist |
| N0233 | Gouty arthritis of the forearm |
| N017. | Helminthiasis with arthropathy |
| N02y8 | Hydroxyapatite deposition disease |
| N0236 | Gouty arthritis of the lower leg |
| N011. | Sexually acquired reactive arthropathy |
| N0167 | Arthropathy associated with mycoses, of the ankle and foot |
| N01wD | Reactive arthropathy of ankle |
| N022. | Chondrocalcinosis, unspecified |
| N015x | Arthropathy with other viral disease, of multiple sites |
| N0234 | Gouty arthritis of the hand |
| N01w0 | Reactive arthropathy of shoulder |
| N01wB | Reactive arthropathy of knee |
| N0163 | Arthropathy associated with mycoses, of the forearm |
| Nyu03 | [X]Other reactive arthropathies |
| N02zK | Crystal arthropathy NOS, of knee |
| N0216 | Chondrocalcinosis-pyrophosphate crystals, of the lower leg |
| N02z. | Crystal arthropathy NOS |
| N0148 | Arthropathy in Whipple's disease |
| N023x | Gouty arthritis of multiple sites |
| N01w5 | Reactive arthropathy of wrist |
| N10y0 | Inflammatory spondylopathies in diseases EC |
| Nyu13 | [X]Other psoriatic arthropathies |
| N023y | Gouty arthritis of other specified site |
| F4642 | Myotonic cataract |
| N01w3 | Reactive arthropathy of elbow |
| N0155 | Arthropathy with other viral disease, of pelvic region/thigh |
| N02y1 | Other crystal arthropathies of the shoulder |
| N012x | Arthropathy in Behcet's syndrome of multiple sites |
| N01wK | Reactive arthropathy of IP joint of toe |
| N0207 | Chondrocalcinosis-dicalcium phosphate, of the ankle and foot |
| N022y | Chondrocalcinosis unspecified, of other specified site |
| N0211 | Chondrocalcinosis-pyrophosphate crystals, of shoulder region |
| N02z3 | Crystal arthropathy NOS, of the forearm |
| N02z6 | Crystal arthropathy NOS, of the lower leg |
| N020. | Chondrocalcinosis due to dicalcium phosphate crystals |
| N02zL | Crystal arthropathy NOS, of tibio-fibular joint |
| N0156 | Arthropathy with other viral disease, of lower leg |
| N021y | Chondrocalcinosis-pyrophosphate crystals, of other spec site |
| N0154 | Arthropathy with other viral disease, of hand |
| N0217 | Chondrocalcinosis-pyrophosphate crystals, of ankle and foot |
| N016. | Arthropathy associated with mycoses |
| N0137 | Postdysenteric reactive arthropathy of the ankle and foot |
| N0157 | Arthropathy with other viral disease, of ankle and foot |
| N012. | Arthropathy in Behcet's syndrome |
| N0153 | Arthropathy with other viral disease, of forearm |
| N01w6 | Reactive arthropathy of MCP joint |
| N0206 | Chondrocalcinosis-dicalcium phosphate, of the lower leg |
| N02z4 | Crystal arthropathy NOS, of the hand |
| N0226 | Chondrocalcinosis unspecified, of the lower leg |
| N0150 | Arthropathy with other viral disease, of unspecified site |
| N0171 | Helminthiasis with arthropathy of the shoulder region |
| N0220 | Chondrocalcinosis unspecified, of unspecified site |
| N02y. | Other crystal arthropathies |
| N02yx | Other crystal arthropathies of multiple sites |
| N021x | Chondrocalcinosis-pyrophosphate crystals, of multiple sites |
| N0214 | Chondrocalcinosis-pyrophosphate crystals, of the hand |
| N011z | Sexually acquired reactive arthropathy NOS |
| N0147 | Arthropathy with other bacterial disease, of ankle and foot |
| N0210 | Chondrocalcinosis-pyrophosphate crystals, of unspec site |
| N0120 | Arthropathy in Behcet's syndrome of unspecified site |
| N0231 | Gouty arthritis of the shoulder region |
| N0140 | Arthropathy with other bacterial disease, of unspec site |
| N0116 | Sexually acquired reactive arthropathy of the lower leg |
| N016z | Arthropathy associated with mycoses NOS |
| N0225 | Chondrocalcinosis unspecified, of the pelvic region/thigh |
| N021z | Chondrocalcinosis due to pyrophosphate crystals, NOS |
| N02z7 | Crystal arthropathy NOS, of the ankle and foot |
| N0238 | Gouty arthritis of toe |
| N0164 | Arthropathy associated with mycoses, of the hand |
| N02y6 | Other crystal arthropathies of the lower leg |
| N02zF | Crystal arthropathy NOS, of PIP joint of finger |
| N0204 | Chondrocalcinosis-dicalcium phosphate, of the hand |
| N01wA | Reactive arthropathy of sacro-iliac joint |
| N02yz | Other crystal arthropathy NOS |

| **Group:** | DMARDs |
| --- | --- |
| **Description:** | Disease-Modifying Antirheumatic Drug |
|  |  |
|  |  |
| **Group:** | **METHOTREXATE_CD** |
| **READ CD** | **Description** |
| h34.. | METHOTREXATE |
| h341. | METHOTREXATE 2.5mg tablets |
| h342. | METHOTREXATE 10mg tablets |
| h343. | METHOTREXATE 2.5mg/1mL solution for injection |
| h344. | METHOTREXATE 5mg/2mL solution for injection |
| h345. | METHOTREXATE 25mg/1mL solution for injection |
| h346. | METHOTREXATE 50mg/2mL solution for injection |
| h347. | METHOTREXATE 100mg/4mL injection solution |
| h348. | METHOTREXATE 200mg/8mL solution for injection |
| h349. | METHOTREXATE 500mg/20mL solution for injection |
| h34A. | METHOTREXATE 15mg/1.5mL solution for injection prefilled syringe |
| h34a. | METHOTREXATE 1g/40mL solution for injection |
| h34B. | METHOTREXATE 20mg/2mL solution for injection prefilled syringe |
| h34b. | METHOTREXATE 5g/200mL solution for injection |
| h34C. | METHOTREXATE 25mg/2.5mL solution for injection prefilled syringe |
| h34c. | METHOTREXATE 500mg injection (pdr for recon) |
| h34D. | METOJECT 7.5mg/0.75mL solution for injection prefilled syringe |
| h34d. | *EMTEXATE 10mg tablets |
| h34E. | METOJECT 10mg/1mL solution for injection prefilled syringe |
| h34e. | EMTEXATE 5mg/2mL solution for injection |
| h34F. | METOJECT 15mg/1.5mL solution for injection prefilled syringe |
| h34f. | EMTEXATE 50mg/2mL solution for injection |
| h34G. | METOJECT 20mg/2mL solution for injection prefilled syringe |
| h34g. | EMTEXATE 250mg/10mL solution for injection |
| h34H. | METOJECT 25mg/2.5mL solution for injection prefilled syringe |
| h34h. | EMTEXATE 500mg/20mL solution for injection |
| h34i. | EMTEXATE 1g/40mL solution for injection |
| h34j. | EMTEXATE 5g/200mL solution for injection |
| h34k. | EMTEXATE 1g/10mL solution for injection |
| h34L. | METOJECT 7.5mg/0.15mL solution for injection pfs |
| h34l. | EMTEXATE 5g/50mL solution for injection |
| h34M. | METHOTREXATE 7.5mg/0.15mL solution for injection pfs |
| h34m. | EMTEXATE 0.5g injection (pdr for recon) |
| h34N. | METOJECT 10mg/0.2mL solution for injection prefilled syringe |
| h34n. | EMTEXATE 1g injection (pdr for recon) |
| h34O. | METHOTREXATE 10mg/0.2mL solution for injection pfs |
| h34o. | EMTEXATE 5g injection (pdr for recon) |
| h34P. | METOJECT 15mg/0.3mL solution for injection prefilled syringe |
| h34p. | MAXTREX 2.5mg tablets |
| h34Q. | METHOTREXATE 15mg/0.3mL solution for injection pfs |
| h34q. | MAXTREX 10mg tablets |
| h34R. | METOJECT 20mg/0.4mL solution for injection prefilled syringe |
| h34r. | MAXTREX 5mg/2mL solution for injection |
| h34S. | METHOTREXATE 20mg/0.4mL solution for injection pfs |
| h34T. | METOJECT 25mg/0.5mL solution for injection prefilled syringe |
| h34U. | METHOTREXATE 25mg/0.5mL solution for injection pfs |
| h34V. | METOJECT 30mg/0.6mL solution for injection prefilled syringe |
| h34W. | METHOTREXATE 30mg/0.6mL solution for injection pfs |
| h34w. | METHOTREXATE 1g/10mL injection |
| h34X. | EBETREX 7.5mg/0.75mL soln for injection prefilled syringe |
| h34x. | METHOTREXATE 5g/50mL injection |
| h34Y. | EBETREX 10mg/1mL solution for injection prefilled syringe |
| h34y. | METHOTREXATE 7.5mg/0.75mL solution for injection prefilled syringe |
| h34Z. | EBETREX 15mg/1.5mL solution for injection prefilled syringe |
| h34z. | METHOTREXATE 10mg/1mL solution for injection prefilled syringe |
| h3G1. | EBETREX 20mg/1mL solution for injection prefilled syringe |
| h3G2. | EBETREX 25mg/1.25mL solution for injection prefilled syringe |
| h3G3. | EBETREX 30mg/1.5mL solution for injection prefilled syringe |
| h3G4. | METHOTREXATE 20mg/1mL solution for injection p/f syringe |
| h3G5. | METHOTREXATE 25mg/1.25mL solution for injection p/f syringe |
| h3G6. | METHOTREXATE 30mg/1.5mL solution for injection p/f syringe |
| h3G7. | METOJECT 12.5mg/0.25mL soln for injection prefilled syringe |
| h3G8. | METHOTREXATE 12.5mg/0.25mL solution for injection pfs |
| h3G9. | METOJECT 17.5mg/0.35mL soln for injection prefilled syringe |
| h3GA. | METHOTREXATE 17.5mg/0.35mL solution for injection pfs |
| h3GB. | METOJECT 22.5mg/0.45mL soln for injection prefilled syringe |
| h3GC. | METHOTREXATE 22.5mg/0.45mL solution for injection pfs |
| h3GD. | METOJECT 27.5mg/0.55mL soln for injection prefilled syringe |
| h3GE. | METHOTREXATE 27.5mg/0.55mL solution for injection pfs |

|  |  |
| --- | --- |
| **Group:** | **LEFLUNOMIDE_CD** |
| **READ CD** | **Description** |
| j59z. | Leflunomide 100mg tablets |
| j59x. | Leflunomide 10mg tablets |
| j59y. | Leflunomide 20mg tablets |
| j591. | Arava 10mg tablets (Sanofi) |
| j592. | Arava 20mg tablets (Sanofi) |
| j593. | Arava 100mg tablets (Sanofi) |
|  |  |
| **Group:** | **SULPHASALAZINE_CD** |
| **READ CD** | **Description** |
| aa6z. | Sulfasalazine 3g/100ml enema |
| aa64. | Salazopyrin 3g/100ml Enema (Pharmacia Ltd) |
| aa62. | Salazopyrin EN-Tabs 500mg (Pfizer Ltd) |
| j551. | Salazopyrin EN-Tabs 500mg (Pfizer Ltd) |
| aa6v. | Sulfasalazine 500mg gastro-resistant tablets |
| aa61. | Salazopyrin 500mg Tablet (Pharmacia Ltd) |
| aa6y. | Sulfasalazine 500mg tablet |
| j55z. | Sulfasalazine 500mg suppositories |
| aa63. | Salazopyrin 500mg Suppository (Pharmacia Ltd) |
| aa6u. | Sulfasalazine 250mg/5ml oral solution |
| aa65. | Salazopyrin 250mg/5ml oral suspension (Pfizer Ltd) |
| aa66. | Sulfasalazine 500mg gastro-resistant tablets (Actavis UK Ltd) |
| j552. | Sulazine EC 500mg tablets (Genesis Pharmaceuticals Ltd) |
|  |  |
|  |  |
| **Group:** | **ANTIMALARIAL_CD** |
| **READ CD** | **Description** |
| ej26. | Chloroquine phosphate 250mg tablets |
| ej25. | Nivaquine 272.5mg(200mg base)/5ml Injection (Aventis Pharma) |
| ejC.. | Chloroquine phosphate 250mg tablets and Proguanil 100mg tablets |
| j54z. | Hydroxychloroquine 200mg tablets |
| ej24. | Nivaquine 68mg/5ml Oral solution (Aventis Pharma) |
| ej23. | Nivaquine 200mg Tablet (Aventis Pharma) |
| ej21. | Avloclor 250mg tablets (AstraZeneca UK Ltd) |
| ej31. | Plaquenil 200mg tablets (Sanofi) |
| j541. | Plaquenil 200mg tablets (Sanofi) |
| ej2w. | Chloroquine phosphate 80mg/5ml oral solution |
| ej22. | Malarivon 80mg/5ml syrup (Wallace Manufacturing Chemists Ltd) |
| j542. | Quinoric 200mg tablets (Bristol Laboratories Ltd) |
|  |  |
| **Group:** | **AZATHIOPRINE_CD** |
| **READ CD** | **Description** |
| h71z. | Azathioprine 50mg powder for solution for injection vials |
| h71y. | Azathioprine 25mg tablets |
| h71x. | Azathioprine 50mg tablets |
| h712. | Imuran 25mg Tablet (Wellcome Medical Division) |
| h713. | Imuran 50mg Tablet (Wellcome Medical Division) |
| h711. | Azamune 50mg Tablet (Penn Pharmaceuticals Ltd) |
| h718. | Azathioprine 10mg tablets |
| h714. | Imuran 50mg powder for solution for injection vials (Aspen Pharma Trading Ltd) |
| h717. | Oprisine 50mg Tablet (Opus Pharmaceuticals Ltd) |
| h715. | Immunoprin 50mg tablets (Ashbourne Pharmaceuticals Ltd) |
| h716. | Berkaprine 50mg Tablet (Rorer Pharmaceuticals Ltd) |
| h719. | Imuran 10mg Tablet (Wellcome Medical Division) |
|  |  |
| **Group:** | **BCell_Antibody_CD** |
| **READ CD** | **Description** |
| hh11. | Rituximab 100mg/10ml solution for infusion vials |
| hh12. | Rituximab 500mg/50ml solution for infusion vials |
|  |  |
| **Group:** | **GOLD_CD** |
| **READ CD** | **Description** |
| j513. | Myocrisin 10mg/0.5ml solution for injection ampoules (Sanofi) |
| j515. | Myocrisin 50mg/0.5ml solution for injection ampoules (Sanofi) |
| j514. | Myocrisin 20mg/0.5ml solution for injection ampoules (Sanofi) |
| j51z. | Sodium aurothiomalate 50mg/0.5ml solution for injection ampoules |
| j51x. | Sodium aurothiomalate 10mg/0.5ml solution for injection ampoules |
| j51y. | Sodium aurothiomalate 20mg/0.5ml solution for injection ampoules |

| **Group:** | PSORIATIC_CD |
| --- | --- |
| **Description:** | Psoriatic arthritis codes |
|  |  |
| **READ CD** | **Description** |
| M160. | Psoriatic arthritis |
| M1600 | Psoriatic arthritis with spine involvement |
| M160z | Psoriatic arthropathy NOS |

| **Group:** | NSAIDs |
| --- | --- |
| **Description:** | Nonsteroidal anti-inflammatory drugs |
|  |  |
| **READ CD** | **Description** |
| j2... | Non-steroidal anti-inflammatory drug |
| j2m.. | Aceclofenac |
| j2m2. | Aceclofenac 100mg tablet |
| j2m1. | Preservex 100mg tablet |
| j2j.. | Acemetacin |
| j2j1. | Acemetacin 60mg capsule |
| j2j2. | Emflex 60mg capsule |
| x02LX | Aspirin |
| x04tL | Oral aspirin |
| bu25. | Aspirin 75mg tablet |
| bu24. | Angettes 75mg tablet |
| bu23. | Aspirin 75mg dispersible tablet |
| bu2D. | postMI 75mg dispersible tablet |
| bu2B. | Aspirin 75mg e/c tablet |
| bu2F. | Caprin 75mg e/c tablet |
| bu2H. | Enprin 75mg e/c tablet |
| bu2K. | Micropirin 75mg e/c tablet |
| bu2A. | Nu-Seals Aspirin 75mg e/c tablet |
| bu2G. | Nu-Seals Cardio 75 e/c tablet |
| bu2E. | postMI 75mg e/c tablet |
| bu2c. | Aspirin 75mg soluble tablet |
| bu21. | Aspirin 100mg effervescent tablet |
| bu22. | Platet 100mg effervescent tablet |
| bu29. | Aspirin 100mg m/r tablet |
| bu28. | Disprin CV 100mg m/r tablet |
| j111. | Aspirin 300mg tablet |
| di15. | Claradin 300mg tablet |
| di16. | Laboprin 300mg tablet |
| di11. | Aspirin [CNS] 300mg tablet |
| j112. | Aspirin 300mg dispersible tablet |
| di1r. | Disprin 300mg dispersible tablet |
| di18. | Solprin 300mg dispersible tablet |
| di12. | Aspirin [CNS] 300mg dispersible tablet |
| bu27. | Aspirin 300mg effervescent tablet |
| bu26. | Platet 300mg effervescent tablet |
| di1m. | Aspirin 300mg soluble tablet |
| di1f. | Aspirin 300mg e/c tablet |
| bu2C. | postMI 300mg e/c tablet |
| di1k. | Caprin 300mg e/c tablet |
| di1c. | Nu-Seals Aspirin 300mg e/c tablet |
| bu2b. | Aspirin 300mg m/r tablet |
| bu2a. | Disprin CV 300mg m/r tablet |
| di1h. | Aspirin 324mg e/c tablet |
| di1a. | Caprin 324mg e/c tablet |
| di19. | Aspirin 500mg m/r tablet |
| x02AA | Anadin All-Night 500mg m/r tablet |
| di1b. | Levius 500mg m/r tablet |
| di1p. | Aspirin 500mg soluble tablet |
| di1q. | Aspro Clear Maximum Strength soluble tablet |
| di1i. | Aspirin 600mg tablet |
| di17. | Paynocil 600mg tablet |
| di1e. | Palaprin Forte 600mg tablet |
| di1g. | Aspirin 600mg e/c tablet |
| di1d. | Nu-Seals Aspirin 600mg e/c tablet |
| bu2I. | Aspirin 162.5mg m/r capsule |
| bu2J. | Caspac XL 162.5mg m/r capsule |
| di14. | Aspergum 227mg chewing gum |
| di1j. | Laboprin DL 900mg sachet |
| x01Kv | Aspirin tablets |
| x01Kw | Rectal Aspirin |
| di1o. | Aspirin 150mg suppository |
| di1n. | Aspirin 300mg suppository |
| x01Kx | Aspirin compound preparation |
| x000x | Co-codaprin |
| dia4. | Co-codaprin 8mg/400mg tablet |
| dia5. | Co-codaprin 8mg/400mg dispersible tablet |
| diab. | Codis dispersible tablet |
| diaO. | Co-codaprin 8mg/500mg dispersible tablet |
| diaP. | Codis 500 dispersible tablet |
| x00et | Aspirin+codeine 300mg/8mg tablet |
| x00I6 | Aspirin+glycine 500mg/133mg dispersible tablet |
| x00I7 | Disprin Direct dispersible tablet |
| dibA. | Aspirin+methocarbamol 325mg/400mg tablet |
| dibk. | Robaxisal Forte tablet |
| diaG. | Aspirin+papaveretum 500mg/7.71mg dispersible tablet |
| dia9. | Aspav dispersible tablet |
| diaE. | Alka-Seltzer tablet |
| diay. | Anadin tablet |
| diaD. | Anadin Extra tablet |
| dia8. | Antoin dispersible tablet |
| diaz. | Askit powder |
| diaB. | Beechams powder |
| diae. | Doloxene Compound capsule |
| diaf. | Equagesic tablet |
| diai. | Hypon tablet |
| dicH. | Phensic tablet |
| dibq. | Trancoprin tablet |
| dia1. | Aspirin/paracetamol/codeine tablets |
| diat. | Aspirin+papaveretum 500mg/10mg dispersible tablet |
| di1.. | Aspirin [central nervous system use] |
| j11.. | Aspirin [musculoskeletal use] |
| j21.. | Azapropazone |
| j21y. | Azapropazone 300mg capsule |
| j211. | Rheumox 300mg capsule |
| j21z. | Azapropazone 600mg tablet |
| j212. | Rheumox 600mg tablet |
| x02Mm | Benorilate |
| j12x. | Benorilate 750mg tablet |
| j121. | Benoral 750mg tablet |
| j12y. | Benorilate 2g/sachet granules |
| j122. | Benoral 2g/sachet granules |
| j12z. | Benorilate 2g/5mL s/f suspension |
| x00eL | Benoral 2g/5mL s/f suspension |
| j123. | Benoral 2g/5mL sugar free suspension 150mL |
| j124. | Benoral 2g/5mL sugar free suspension 300mL |
| di3.. | Benorylate [central nervous sytem use] |
| j12.. | Benorilate [musculoskeletal use] |
| j2t.. | Dexibuprofen |
| j2ty. | Dexibuprofen 300mg tablet |
| j2t2. | Seractil 300mg tablet |
| j2tz. | Dexibuprofen 400mg tablet |
| j2t1. | Seractil 400mg tablet |
| j2q.. | Dexketoprofen |
| j2qz. | Dexketoprofen 25mg tablet |
| j2q1. | Keral 25mg tablet |
| x01Ky | Diclofenac |
| x01Kz | Oral diclofenac |
| j2ry. | Diclofenac potassium 25mg tablet |
| j2r1. | Voltarol Rapid 25mg tablet |
| j2rz. | Diclofenac potassium 50mg tablet |
| j2r2. | Voltarol Rapid 50mg tablet |
| j22d. | Diclofenac sodium 25mg e/c tablet |
| j2oC. | Acoflam 25mg e/c tablet |
| j2oo. | Defanac 25mg e/c tablet |
| j22I. | Dicloflex 25mg e/c tablet |
| j2o4. | Diclovol 25mg e/c tablet |
| j22l. | Diclozip-25 e/c tablet |
| j2oq. | Fenactol 25mg e/c tablet |
| j22w. | Flamrase 25mg e/c tablet |
| j22L. | Lofensaid 25 tablet |
| j227. | Rhumalgan 25mg e/c tablet |
| j22j. | Valenac 25mg e/c tablet |
| j22f. | Volraman 25mg e/c tablet |
| x00LD | Voltarene 25mg e/c tablet |
| j221. | Voltarol 25mg e/c tablet |
| j22u. | Diclofenac sodium 25mg tablets |
| j22e. | Diclofenac sodium 50mg e/c tablet |
| j2oB. | Acoflam 50mg e/c tablet |
| j2op. | Defanac 50mg e/c tablet |
| j22J. | Dicloflex 50mg e/c tablet |
| j2o5. | Diclovol 50mg e/c tablet |
| j22m. | Diclozip-50 e/c tablet |
| j2or. | Fenactol 50mg e/c tablet |
| j22x. | Flamrase 50mg e/c tablet |
| j22s. | Isclofen 50mg e/c tablet |
| j22M. | Lofensaid 50 tablet |
| j228. | Rhumalgan 50mg e/c tablet |
| j22k. | Valenac 50mg e/c tablet |
| j22g. | Volraman 50mg e/c tablet |
| x00LE | Voltarene 50mg e/c tablet |
| j222. | Voltarol 50mg e/c tablet |
| j22v. | Diclofenac sodium 50mg tablets |
| j22i. | Diclofenac sodium 50mg dispersible tablet |
| j22h. | Voltarol 50mg dispersible tablet |
| j22y. | Diclofenac sodium 75mg m/r tablet |
| j2oA. | Acoflam SR 75mg m/r tablet |
| j2ol. | Defanac SR 75mg m/r tablet |
| j2o8. | Dexomon SR 75mg m/r tablet |
| j2o1. | Dicloflex SR 75mg m/r tablet |
| j2o6. | Diclovol SR 75mg m/r tablet |
| j22Y. | Diclotard 75 m/r tablet |
| j2ow. | Econac SR 75mg tablet |
| j2os. | Fenactol SR 75mg m/r tablet |
| j2o2. | Flamatak MR 75mg m/r tablet |
| j2oG. | Flamrase SR 75mg m/r tablet |
| j2oF. | Rheumatac Retard 75mg m/r tablet |
| j22O. | Rhumalgan CR 75mg m/r tablet |
| j22S. | Lofensaid Retard 75 m/r tablet |
| j22V. | Slofenac SR 75mg m/r tablet |
| j2ou. | Valdic 75 Retard 75mg m/r tablet |
| j22P. | Volsaid Retard 75mg m/r tablet |
| j22n. | Voltarol SR 75mg m/r tablet |
| j22C. | Diclofenac sodium 75mg m/r capsule |
| j22H. | Diclomax SR 75mg m/r capsule |
| j2oM. | Rhumalgan SR 75mg m/r capsule |
| j22R. | Diclofenac sodium 75mg e/c+m/r capsule |
| j22B. | Motifene 75mg e/c+m/r capsule |
| j22c. | Diclofenac sodium 100mg m/r tablet |
| j2oD. | Acoflam Retard 100mg m/r tablet |
| j2oJ. | Closteril 100 m/r tablet |
| j2om. | Defanac Retard 100mg m/r tablet |
| j2o9. | Dexomon Retard 100mg m/r tablet |
| j22K. | Dicloflex Retard 100mg m/r tablet |
| j22Z. | Diclotard 100 m/r tablet |
| j2o7. | Diclovol Retard 100mg m/r tablet |
| j2o3. | Difenor XL 100mg m/r tablet |
| j22W. | Digenac XL 100 m/r tablet |
| j2ox. | Econac XL 100mg tablet |
| j2ot. | Fenactol Retard 100mg m/r tablet |
| j2oE. | Flamatak MR 100mg m/r tablet |
| j22A. | Flamrase SR 100mg m/r tablet |
| j22X. | Flexotard MR 100mg m/r tablet |
| j22T. | Lofensaid Retard 100 m/r tablet |
| j22N. | Rhumalgan CR 100mg m/r tablet |
| j22U. | Slofenac SR 100mg m/r tablet |
| j2ov. | Valdic 100 Retard 100mg m/r tablet |
| j22Q. | Volsaid Retard 100mg m/r tablet |
| x00Ts | Voltaren Retard 100mg m/r tablet |
| x00Tt | Voltarene LP 100mg m/r tablet |
| j226. | Voltarol Retard 100mg m/r tablet |
| j22r. | Diclofenac sodium 100mg m/r capsule |
| j22q. | Diclomax Retard 100mg m/r capsule |
| j2oL. | Rhumalgan XL 100mg m/r capsule |
| x01L0 | Rectal diclofenac |
| j22a. | Diclofenac sodium 100mg suppository |
| j2oH. | Econac 100mg suppository |
| j224. | Voltarol 100mg suppository |
| j22F. | Diclofenac sodium 25mg suppository |
| j22D. | Voltarol 25mg suppository |
| j22G. | Diclofenac sodium 50mg suppository |
| j22E. | Voltarol 50mg suppository |
| j22b. | Diclofenac sodium 12.5mg paediatric suppository |
| j225. | Voltarol 12.5mg paediatric suppository |
| x01L1 | Parenteral diclofenac |
| j229. | Diclofenac sodium 75mg/3mL injection |
| j2oK. | Econac 75mg/3mL injection |
| j223. | Voltarol 75mg/3mL injection |
| j2oO. | DICLOFENAC 75mg/2mL solution for injection |
| j2oN. | DYLOJECT 75mg/2mL solution for injection |
| x00xb | Topical diclofenac |
| ja1Y. | Diclofenac sodium 1% gel |
| x00C5 | Voltarol Emulgel |
| ja1A. | Voltarol Emulgel Topical gel 100g |
| ja1F. | Voltarol Emulgel Topical gel 20g |
| x05d3 | Voltarol Emulgel P topical gel |
| ja2g. | Voltarol Emulgel P topical gel 30g |
| ja2m. | Voltarol Emulgel P topical gel 50g |
| x05wX | Voltarol Pain-eze Emulgel 1% topical gel |
| ja2q. | Voltarol Pain-eze Emulgel 1% topical gel 30g |
| ja2w. | Diclofenac 1% 10cmx14cm patch |
| ja2s. | Voltarol Gel 1% 10cmx14cm patch |
| mt1z. | Diclofenac sodium 3% gel |
| x05J3 | Solaraze 3% gel |
| mt11. | Solaraze 3% gel 25g |
| ja2y. | Diclofenac sodium 1.5% topical solution |
| x05FX | Pennsaid 16mg/mL cutaneous solution |
| ja2e. | Pennsaid 1.5% topical solution 60mL |
| k6d5. | Diclofenac sodium 0.1% single-use eye drops |
| x00ca | Voltarol Ophtha 0.1% single-use eye drops |
| k6d1. | Voltarol Ophtha 0.1% single-use eye drops x4 |
| k6d2. | Voltarol Ophtha 0.1% single-use eye drops x40 |
| k6d6. | Voltarol Ophtha 0.1% single-use eye drops x5 |
| k6d3. | Diclofenac 0.1% single-use eye drops x4 |
| k6d4. | Diclofenac 0.1% single-use eye drops x40 |
| k6d7. | Diclofenac sodium 0.1% multidose eye drops |
| x05lh | Voltarol Ophtha 0.1% multidose eye drops |
| k6d8. | Voltarol Ophtha 0.1% multidose eye drops 5mL |
| j22.. | Diclofenac sodium product |
| j2o.. | Diclofenac sodium 2 |
| j2oP. | VOLTAROL ACTIVE 4% spray 15mL |
| j2oQ. | VOLTAROL ACTIVE 4% spray 30mL |
| j2oR. | ENSTAR XL 100mg m/r tablets |
| j2oy. | MOBIGEL 4% spray 25g |
| j2oz. | DICLOFENAC SODIUM 4% spray |
| j2r.. | Diclofenac potassium |
| j2r3. | VOLTAROL PAIN-EZE 12.5mg tablets |
| j2rx. | DICLOFENAC POTASSIUM 12.5mg tablets |
| k6d.. | Diclofenac sodium [eye] |
| mt1.. | Diclofenac sodium [actinic keratosis] |
| mt12. | SOLARAZE 3% gel 50g |
| mt13. | SOLARAZE 3% gel 100g |
| x02MM | Diflunisal |
| j233. | Diflunisal 250mg tablet |
| j231. | Dolobid 250mg tablet |
| x00fB | Dolobis 250mg tablet |
| j234. | Diflunisal 500mg tablet |
| j232. | Dolobid 500mg tablet |
| di4.. | Diflunisal [CNS analgesic] [see j23..] |
| j23.. | Diflunisal [musculoskeletal use] |
| j24.. | Etodolac |
| j246. | Etodolac 200mg tablet |
| j245. | Lodine 200mg tablet |
| j242. | Ramodar 200mg tablet |
| j243. | Etodolac 200mg capsule |
| j24A. | Ebretin 200mg capsule |
| j241. | Lodine 200mg capsule |
| j247. | Etodolac 300mg capsule |
| j24B. | Ebretin 300mg capsule |
| j24C. | Eccoxolac 300mg capsule |
| j244. | Lodine 300mg capsule |
| j249. | Etodolac 600mg m/r tablet |
| j24D. | ETOPAN XL 600mg m/r tablets |
| j248. | Lodine SR 600mg m/r tablet |
| j25.. | Fenbufen |
| j258. | Fenbufen 300mg tablet |
| j25A. | Fenbuzip 300mg tablet |
| j253. | Lederfen 300mg tablet |
| j254. | Lederfen CP 300mg tablets |
| j257. | Fenbufen 300mg capsule |
| x00Dx | Cinopal 300mg capsule |
| j259. | Fenbuzip 300mg capsule |
| x026C | Lederfen 300mg capsule |
| j251. | Lederfen 300mg capsules x21 |
| j252. | Lederfen CP 300mg capsules x84 |
| j25y. | Fenbufen 450mg tablet |
| j25B. | Fenbuzip 450mg tablet |
| j255. | Lederfen 450mg tablet |
| j25z. | Fenbufen 450mg effervescent tablet |
| x026D | Lederfen F 450mg effervescent tablet |
| j256. | Lederfen F 450mg effervescent tablets 56 |
| x01L2 | Felbinac |
| ja1u. | Felbinac 3% gel |
| x026W | Traxam gel |
| ja1S. | Traxam 3% topical gel 100g |
| ja1v. | Traxam 3% topical gel 50g |
| x03hC | Traxam Pain Relief gel |
| ja2K. | Traxam Pain Relief gel 30g |
| ja1T. | Felbinac 3.17% foam |
| x026G | Traxam foam |
| ja1K. | TRAXAM 3.17% foam 100g |
| x02MN | Fenoprofen |
| di5z. | Fenoprofen 200mg tablet |
| di51. | Progesic 200mg tablet |
| j263. | Fenoprofen 300mg tablet |
| j261. | Fenopron 300mg tablet |
| j264. | Fenoprofen 600mg tablet |
| j262. | Fenopron 600mg tablet |
| x00fF | Fepron 600mg tablet |
| di5.. | Fenoprofen [analgesic] |
| j26.. | Fenoprofen [musculoskeletal use] |
| j27.. | Flurbiprofen |
| j27x. | Flurbiprofen 50mg tablet |
| j271. | Froben 50mg tablet |
| j27y. | Flurbiprofen 100mg tablet |
| j272. | Froben 100mg tablet |
| j275. | Flurbiprofen 200mg m/r capsule |
| j274. | Froben SR 200mg m/r capsule |
| lf3z. | Flurbiprofen 8.75mg lozenge |
| lf33. | Strefen Honey and Lemon 8.75mg lozenge |
| lf31. | Strefen 8.75mg lozenge |
| lf32. | Streflam 8.75mg lozenge |
| j27z. | Flurbiprofen 100mg suppository |
| j273. | Froben 100mg suppository |
| x026y | Flurbiprofen sodium 0.03% single-use eye drops |
| x026x | Ocufen 0.03% single-use eye drops |
| k6c1. | Ocufen 0.03% single-use eye drops 0.4mL |
| k6cz. | Flurbiprofen sodium 0.03% single-use eye drops 0.4mL |
| k6c.. | Flurbiprofen sodium [eye] |
| lf3.. | Flurbiprofen [oropharyngeal] |
| x02MO | Ibuprofen product |
| x01L3 | Oral ibuprofen |
| j281. | Ibuprofen 200mg tablet |
| j28W. | Advil 200mg tablet |
| j28Z. | Anadin Ibuprofen 200mg tablet |
| j284. | Apsifen 200mg tablet |
| j28q. | Arthrofen 200 tablet |
| j287. | Brufen 200mg tablet |
| j28C. | Cuprofen 200mg tablet |
| j28b. | Ebufac 200mg tablet |
| j2pQ. | Galprofen Ibuprofen 200mg Caplet |
| x00fN | Ibrufhalal 200mg tablet |
| j2pZ. | IBUCALM 200mg tablets |
| j28e. | Ibular 200mg tablet |
| j28g. | Ibumetin 200mg tablet |
| j28R. | Inoven 200mg Caplet |
| j2p5. | Librofem 200mg tablet |
| j28E. | Lidifen 200mg tablet |
| x00fZ | Migrafen 200mg tablet |
| j28j. | Motrin 200mg tablet |
| j2pH. | Nurofen 200mg tablet |
| j2p1. | Nurofen Advance 200mg tablet |
| j2pI. | Nurofen 200mg Caplet |
| j2pD. | Nurofen Mobile 200mg tablet |
| j2pS. | Nurofen Tension Headache 200mg tablet |
| x00fi | Pacifene 200mg tablet |
| j28m. | Paxofen 200mg tablet |
| x00fn | Phor Pain 200mg tablet |
| x00fs | Proflex 200mg tablet |
| j28S. | Relcofen 200mg tablet |
| j28M. | Rimafen 200mg tablet |
| j282. | Ibuprofen 400mg tablet |
| j28X. | Advil Extra Strength 400mg tablet |
| j285. | Apsifen 400mg tablet |
| j28r. | Arthrofen 400 tablet |
| j288. | Brufen 400mg tablet |
| j28Q. | Cuprofen Maximum Strength 400mg tablet |
| j28c. | Ebufac 400mg tablet |
| j2pa. | IBUCALM 400mg tablets |
| j28K. | Ibumed 400mg tablet |
| j28h. | Ibumetin 400mg tablet |
| j28f. | Ibular 400mg tablet |
| j28D. | Isisfen 400mg tablet |
| j28F. | Lidifen 400mg tablet |
| j2pB. | Mandafen 400mg tablet |
| j28k. | Motrin 400mg tablet |
| j2pU. | Nurofen Maximum Strength Migraine Pain 400mg Caplet |
| x00fh | Pacifene 400mg tablet |
| j28n. | Paxofen 400mg tablet |
| x00fm | Phor Pain 400mg tablet |
| j28N. | Rimafen 400mg tablet |
| j28T. | Relcofen 400mg tablet |
| j283. | Ibuprofen 600mg tablet |
| j28L. | Apsifen F 600mg tablet |
| j286. | Apsifen F 600mg effervescent tablets |
| j28t. | Arthrofen 600 tablet |
| j289. | Brufen 600mg tablet |
| j28i. | Ibumetin 600mg tablet |
| j28G. | Lidifen 600mg tablet |
| j2p7. | Mandafen 600mg tablet |
| j28l. | Motrin 600mg tablet |
| j28o. | Paxofen 600mg tablet |
| j28u. | Ibuprofen 800mg tablet |
| j28p. | Motrin 800mg tablet |
| j2pJ. | Ibuprofen 200mg capsule |
| diaW. | Anadin Ultra 200mg capsule |
| j2pK. | Nurofen 200mg liquid capsule |
| j28x. | Ibuprofen 200mg m/r capsule |
| j28y. | Ibuprofen 300mg m/r capsule |
| j28d. | Fenbid 300mg Spansule |
| j2p3. | Nurofen Back Pain SR 300mg m/r capsule |
| x00Tg | Proflex 300mg m/r capsule |
| j2pW. | IBUPROFEN 400mg capsules |
| j2pV. | NUROFEN EXTRA STRENGTH 400mg liquid capsules |
| j2pY. | GALPHARM MAXIMUM STRENGTH IBUPROFEN 400mg liquid capsules |
| j28B. | Ibuprofen 800mg m/r tablet |
| j28A. | Brufen Retard 800mg m/r tablet |
| j28w. | Ibuprofen 100mg/5mL oral suspension |
| j28a. | Brufen 100mg/5mL syrup |
| j28Y. | Ibuprofen 100mg/5mL s/f oral suspension |
| j2pL. | Calprofen 100mg/5mL s/f oral suspension |
| j2pO. | Care Ibuprofen for Children 100mg/5mL s/f oral suspension |
| j2pG. | Cuprofen For Children 100mg/5mL s/f oral suspension |
| j2pE. | Fenpaed 100mg/5mL s/f oral suspension |
| j2p8. | Feverfen 100mg/5mL s/f oral suspension |
| j2p4. | Galprofen 100mg/5mL oral suspension |
| j28J. | Junifen 100mg/5mL s/f suspension |
| j28s. | Junifen 100mg/5mL suspension 150mL |
| j2pR. | Lloyds Pharmacy Ibuprofen for Children 100mg/5mL s/f oral suspension |
| j2pC. | Orbifen 100mg/5mL s/f oral suspension |
| j2p6. | Orbifen For Children 100mg/5mL s/f oral suspension |
| j2pF. | Mandafen For Children 100mg/5mL s/f oral suspension |
| j2p2. | Nurofen For Children 100mg/5mL oral suspension |
| j2pP. | Nurofen For Children Strawberry 100mg/5mL s/f oral suspension |
| j2pA. | Ibuprofen 100mg/5mL sachets s/f oral suspension |
| j2p9. | Nurofen For Children Singles 100mg/5mL sachets s/f oral suspension |
| j2pN. | Orbifen 100mg/5mL s/f oral suspension sachets |
| j28P. | Ibuprofen 600mg/sachet effervescent granules |
| j28v. | Brufen 600mg/sachet effervescent granules |
| x006P | Ibuprofen+codeine phosphate |
| j28V. | Ibuprofen+codeine phosphate 200mg/12.8mg tablet |
| j2pM. | Cuprofen PLUS tablet |
| j2pT. | Solpadeine Migraine Ibuprofen & Codeine tablet |
| j28U. | Solpaflex tablet |
| j28I. | Nurofen Plus tablet |
| j28z. | Ibuprofen+codeine phosphate 300mg/20mg m/r tablet |
| j28H. | Codafen Continus m/r tablet |
| j28O. | Ibuprofen+codeine phosphate 200mg/12.5mg tablet |
| x05YT | Oral ibuprofen compound product |
| x05YU | Ibuprofen+pseudoephedrine hydrochloride |
| dic6. | Ibuprofen+pseudoephedrine 200mg/30mg tablet |
| dic7. | Nurofen Cold & Flu tablet |
| dic8. | Nurofen Sinus tablet |
| dibC. | Sudafed Dual Relief Max tablet |
| dicC. | Ibuprofen+pseudoephedrine 300mg/45mg capsule |
| dicB. | Lemsip Flu 12HR Ibuprofen+Pseudoephedrine capsule |
| dicP. | Lemsip Cold & Flu Sinus 12 Hr Ibuprofen + Pseudoephedrine 300mg/45mg capsule |
| j2pX. | ANADIN ULTRA DOUBLE STRENGTH 400mg liquid capsules |
| x01L4 | Topical ibuprofen |
| ja1x. | Ibuprofen 5% cream |
| x00HG | Proflex cream |
| ja1J. | Proflex 5% cream 50g |
| ja1w. | Proflex 5% cream 100g |
| x01L5 | Proflex Pain Relief cream |
| ja1R. | Proflex pain relief 5% cream 25g |
| ja22. | Proflex pain relief 5% cream 30g |
| ja2U. | Ibuprofen 5% foam |
| x03wA | Ibuleve 5% mousse |
| ja2V. | Ibuleve 5% mousse 125g |
| ja2a. | Ibuleve 5% mousse 75g |
| x04vt | Ibumousse 5% foam |
| ja2W. | Ibumousse 5% foam 125g |
| ja1U. | Ibuprofen 5% gel |
| x049k | Cuprofen 5% gel |
| ja2Q. | Cuprofen 5% gel 30g |
| ja2R. | Cuprofen 5% gel 50g |
| x03dl | Fenbid 5% gel |
| ja2H. | Fenbid 5% gel 100g |
| ja2M. | Fenbid 5% gel 30g |
| ja2N. | Fenbid 5% gel 50g |
| x026Q | Ibugel gel |
| ja1I. | Ibugel gel 100g |
| ja1W. | Ibuleve gel |
| x00Ms | Ibuleve Sports gel |
| x04yw | Nurofen gel |
| ja2Z. | Nurofen gel 35g |
| x05q2 | Phorpain 5% gel |
| ja2l. | Phorpain 5% gel 100g |
| ja2z. | Ibuprofen 10% gel |
| x05vE | Care Ibuprofen 10% gel |
| ja2o. | Care Ibuprofen 10% gel 50g |
| x04xv | Fenbid Forte 10% gel |
| ja2X. | Fenbid Forte 10% gel 100g |
| x0588 | Ibugel Forte 10% gel |
| ja2b. | Ibugel Forte 10% gel 100g |
| x05Bt | Ibuleve Maximum Strength 10% gel |
| ja2d. | Ibuleve Maximum Strength 10% gel 30g |
| ja2h. | Ibuleve Maximum Strength 10% gel 50g |
| x05wW | Lloyds Pharmacy Maximum Strength Ibuprofen 10% gel |
| ja2p. | Lloyds Pharmacy Maximum Strength Ibuprofen 10% gel 30g |
| x05Mg | Nurofen Maximum Strength 10% gel |
| ja2f. | Nurofen Maximum Strength 10% gel 30g |
| x05gD | Phorpain Gel Maximum Strength 10% gel |
| ja2i. | Phorpain Gel Maximum Strength 10% gel 30g |
| x02Dr | Ibuprofen+menthol 5%/3% gel |
| x02Ds | Deep Relief Ibuprofen gel |
| ja2A. | Deep Relief Ibuprofen gel 100g |
| ja2F. | Deep Relief Ibuprofen gel 15g |
| ja2G. | Deep Relief Ibuprofen gel 50g |
| ja24. | Ibuprofen 5% spray |
| ja25. | Ibuleve 5% topical spray |
| ja23. | Ibuspray topical spray |
| di6.. | Ibuprofen [cns analgesic] [see j28..] |
| j28.. | Ibuprofen [musculoskeletal use] |
| j2p.. | Ibuprofen [musculoskeletal use 2] |
| j2pb. | NUROFEN EXPRESS SOLUBLE 400mg/sachet oral powder |
| j2pc. | IBUPROFEN 400mg/sachet oral powder |
| j29.. | Indometacin product |
| j291. | Indometacin 25mg capsule |
| j294. | Artracin 25mg capsule |
| j296. | Imbrilon 25mg capsule |
| j299. | Indocid 25mg capsule |
| j29e. | Indoflex 25mg capsule |
| j29f. | Indolar 25mg capsule |
| j29r. | Indomax 25 capsule |
| j29l. | Mobilan 25mg capsule |
| j29F. | Rimacid 25mg capsule |
| j292. | Indometacin 50mg capsule |
| j295. | Artracin 50mg capsule |
| j297. | Imbrilon 50mg capsule |
| j29a. | Indocid 50mg capsule |
| j29g. | Indolar 50mg capsule |
| j29m. | Mobilan 50mg capsule |
| j29E. | Indometacin 25mg m/r tablet |
| j29B. | Flexin-25 Continus m/r tablet |
| j29z. | Indometacin 25mg m/r capsule |
| j29j. | Indomod 25mg m/r capsule |
| j29D. | Indometacin 50mg m/r tablet |
| j29C. | Flexin-50 Continus m/r tablet |
| j29A. | Indometacin 75mg m/r tablet |
| j29p. | Flexin-75 Continus m/r tablet |
| j29y. | Indometacin 75mg m/r capsule |
| j29G. | Artracin SR 75mg m/r capsule |
| j29u. | Berlind 75 Retard 75mg m/r capsule |
| j29d. | Indocid R 75mg m/r capsule |
| j29i. | Indolar SR 75mg m/r capsule |
| j29s. | Indomax 75 SR m/r capsule |
| j29k. | Indomod 75mg m/r capsule |
| j29J. | Indotard MR 75mg m/r capsule |
| j29H. | Maximet SR 75mg m/r capsule |
| j29K. | Pardelprin MR 75mg m/r capsule |
| j29t. | Rheumacin LA 75mg m/r capsule |
| j29o. | Slo-Indo 75mg m/r capsule |
| j29w. | Indomethacin 25mg/5mL s/f suspension |
| j29b. | Indocid 25mg/5mL s/f suspension |
| j293. | Indometacin 100mg suppository |
| j298. | Imbrilon 100mg suppository |
| j29c. | Indocid 100mg suppository |
| j29h. | Indolar 100mg suppository |
| j29v. | Indometacin 1mg injection (pdr for recon) |
| j29q. | Indocid PDA 1mg injection (pdr for recon) |
| j2a.. | Ketoprofen |
| j2a1. | Ketoprofen 50mg capsule |
| j2a4. | Alrheumat 50mg capsule |
| j2aE. | Ketonal 50mg capsule |
| j2a5. | Orudis 50mg capsule |
| j2ab. | Tiloket 50mg capsule |
| j2a2. | Ketoprofen 100mg capsule |
| j2aF. | Ketonal 100mg capsule |
| j2a6. | Orudis 100mg capsule |
| j2ay. | Ketoprofen 100mg m/r capsule |
| j2aQ. | Tiloket CR 100mg m/r capsule |
| j2aA. | Ketoprofen CR 100mg m/r capsule |
| j2aC. | Ketovail 100mg m/r capsule |
| j2aO. | Ketpron XL 100mg m/r capsule |
| j2a8. | Oruvail 100 m/r capsule |
| j2aw. | Ketoprofen 150mg m/r capsule |
| j2aG. | Oruvail 150 m/r capsule |
| j2az. | Ketoprofen 200mg m/r capsule |
| j2aJ. | Fenoket 200mg m/r capsule |
| j2aM. | Jomethid XL 200mg m/r capsule |
| j2aR. | Tiloket CR 200mg m/r capsule |
| j2aI. | Ketocid 200 m/r capsule |
| j2aB. | Ketoprofen CR 200mg m/r capsule |
| j2aL. | Ketotard 200 XL m/r capsule |
| j2aD. | Ketovail 200mg m/r capsule |
| j2aK. | Ketozip CR 200mg m/r capsule |
| j2aP. | Ketpron XL 200mg m/r capsule |
| j2aH. | Larafen CR 200mg m/r capsule |
| j2a9. | Oruvail 200 m/r capsule |
| j2a3. | Ketoprofen 100mg suppository |
| j2a7. | Orudis 100mg suppository |
| j2ax. | Ketoprofen 100mg/2mL injection |
| j2aa. | Oruvail IM 100mg/2mL injection |
| ja1V. | Ketoprofen 2.5% gel |
| x04yU | Tiloket 2.5% gel |
| ja2Y. | Tiloket 2.5% gel 100g |
| ja2r. | Tiloket 2.5% gel 50g |
| x00N4 | Oruvail gel |
| ja1H. | Oruvail gel 100g |
| ja1O. | Oruvail gel 30g |
| ja26. | Powergel 2.5% gel |
| ja28. | Powergel 2.5% gel 50g |
| ja29. | Powergel 2.5% gel 100g |
| ja2J. | Powergel 2.5% gel 50gx2 |
| ja31. | Powergel 2.5% dispenser gel 50g |
| ja32. | Powergel 2.5% dispenser gel 100g |
| x03hs | Solpaflex gel |
| ja2L. | Solpaflex gel 30g |
| j2aS. | VALKET 200 RETARD 200mg m/r capsules |
| j2ac. | AXORID 100mg/20mg m/r capsules |
| j2ad. | AXORID 200mg/20mg m/r capsules |
| j2au. | KETOPROFEN+OMEPRAZOLE 200mg/20mg m/r capsules |
| j2av. | KETOPROFEN+OMEPRAZOLE 100mg/20mg m/r capsules |
| x01L6 | Ketorolac |
| x03ec | Oral ketorolac |
| o4a3. | Ketorolac trometamol 10mg tablet |
| o4a4. | Toradol 10mg tablet |
| x03ed | Parenteral ketorolac |
| o4a5. | Ketorolac trometamol 10mg/1mL injection |
| o4a6. | Toradol 10mg/1mL injection |
| o4a1. | Ketorolac trometamol 30mg/1mL injection |
| o4a2. | Toradol 30mg/1mL injection |
| x03eb | Topical ketorolac |
| k6g1. | Ketorolac trometamol 0.5% ophthalmic solution |
| x03ea | Acular 0.5% ophthalmic solution |
| k6g2. | Acular 0.5% ophthalmic solution 10mL |
| k6g.. | Ketorolac trometamol [eye] |
| o4a.. | Ketorolac trometamol |
| j2s.. | Lornoxicam |
| j2sx. | Lornoxicam 8.6mg injection (pdr for recon) |
| j2s3. | Xefo 8.6mg injection (pdr for recon) |
| j2sz. | Lornoxicam 4mg tablet |
| j2s1. | Xefo 4mg tablet |
| j2sy. | Lornoxicam 8mg tablet |
| j2s2. | Xefo 8mg tablet |
| x02MP | Mefenamic acid |
| j2b1. | Mefenamic acid 250mg capsule |
| j2b8. | Contraflam 250mg capsule |
| j2b6. | Dysman-250 capsule |
| j2b9. | Meflam 250 capsule |
| j2bB. | Opustan 250mg capsule |
| j2b2. | Ponstan 250mg capsule |
| j2bz. | Mefenamic acid 250mg dispersible tablet |
| j2b5. | Ponstan Dispersible 250mg tablet |
| j2bx. | Mefenamic acid 500mg tablet |
| j2bD. | Contraflam 500mg tablet |
| j2b7. | Dysman-500 tablet |
| j2bA. | Meflam 500 tablet |
| j2bC. | Opustan 500mg tablet |
| j2b3. | Ponstan Forte 500mg tablet |
| j2by. | Mefenamic acid 50mg/5mL suspension |
| j2b4. | Ponstan 50mg/5mL paediatric suspension |
| di7.. | Mefenamic acid [cns analgesic] [see j2b..] |
| j2b.. | Mefenamic acid [musculoskeletal use] |
| j2k.. | Nabumetone |
| j2k2. | Nabumetone 500mg tablet |
| j2k1. | Relifex 500mg tablet |
| j2k6. | Nabumetone 500mg dispersible tablet |
| j2k5. | Relifex 500mg dispersible tablet |
| j2k4. | Nabumetone 500mg/5mL suspension |
| j2k3. | Relifex 500mg/5mL suspension |
| j2c.. | Naproxen |
| j2c1. | Naproxen 250mg tablet |
| j2cc. | Arthrosin 250 tablet |
| j2ce. | Arthroxen 250mg tablet |
| j2c3. | Laraflex 250mg tablet |
| j2c5. | Naprosyn 250mg tablet |
| j2cm. | Prosaid 250mg tablet |
| j2cA. | Rheuflex-250 tablet |
| j2cH. | Rimoxyn 250mg tablet |
| j2cI. | Timpron 250mg tablet |
| j2ca. | Valrox 250mg tablet |
| j2ch. | Naproxen 375mg tablet |
| j2cg. | Naprosyn 375mg tablet |
| j2c2. | Naproxen 500mg tablet |
| j2cd. | Arthrosin 500 tablet |
| j2cf. | Arthroxen 500mg tablet |
| j2c4. | Laraflex 500mg tablet |
| j2c6. | Naprosyn 500mg tablet |
| j2cn. | Prosaid 500mg tablet |
| j2cB. | Rheuflex-500 tablet |
| j2cG. | Rimoxyn 500mg tablet |
| j2cF. | Timpron 500mg tablet |
| j2cb. | Valrox 500mg tablet |
| j2ck. | Naproxen 250mg e/c tablet |
| j2cM. | Arthrosin 250mg e/c tablet |
| j2co. | Naprosyn EC 250mg e/c tablet |
| j2ci. | Nycopren 250mg e/c tablet |
| j2cJ. | Timpron 250 EC e/c tablet |
| j2cw. | Naproxen 375mg e/c tablet |
| j2cp. | Naprosyn EC 375mg e/c tablet |
| j2cl. | Naproxen 500mg e/c tablet |
| j2cN. | Arthrosin 500mg e/c tablet |
| j2cq. | Naprosyn EC 500mg e/c tablet |
| j2cj. | Nycopren 500mg e/c tablet |
| j2cK. | Timpron 500 EC e/c tablet |
| j2cs. | Naproxen 375mg m/r tablet |
| j2cC. | Pranoxen Continus 375mg m/r tablet |
| j2ct. | Naproxen 500mg m/r tablet |
| j2cE. | Naprosyn SR 500mg m/r tablet |
| j2cD. | Pranoxen Continus 500mg m/r tablet |
| di8z. | Naproxen sodium 275mg tablet |
| di81. | Synflex 275mg tablet |
| j2cv. | Naproxen 500mg/sachet granules |
| j2c9. | Naprosyn 500mg/sachet granules |
| j2cy. | Naproxen 125mg/5mL suspension |
| j2c7. | Naprosyn 125mg/5mL suspension |
| j2cz. | Naproxen 500mg suppository |
| j2c8. | Naprosyn 500mg suppository |
| di8.. | Naproxen sodium |
| j2cO. | FEMINAX ULTRA 250mg tablets |
| j2cP. | VIMOVO 500mg/20mg m/r tablets |
| j2cZ. | NAPROXEN+ESOMEPRAZOLE 500mg/20mg m/r tablets |
| x01L7 | Nefopam |
| di9y. | Nefopam hydrochloride 30mg tablet |
| di91. | Acupan 30mg tablet |
| di9z. | Nefopam hydrochloride 20mg/1mL injection |
| di92. | Acupan 20mg/1mL injection |
| di9.. | Nefopam hydrochloride |
| k67.. | Oxyphenbutazone |
| x01L8 | Tanderil Chloramphenicol eye ointment |
| k672. | Tanderil chloramphenicol eye ointment 5g |
| k67y. | Oxyphenbutazone 10% eye ointment |
| x00LU | Tanderil eye ointment |
| k671. | Tanderil 10% eye ointment 5g |
| k67z. | Oxyphenbutazone eye ointment |
| j2d.. | Phenylbutazone product |
| j2d1. | Phenylbutazone 100mg tablet |
| j2d5. | Butazolidin 100mg tablet |
| j2d7. | Butazone 100mg tablet |
| x00LC | Phenylbutazone 100mg e/c tablet |
| j2d3. | Butacote 100mg e/c tablet |
| j2d2. | Phenylbutazone 200mg tablet |
| j2d4. | Butacote 200mg tablet |
| j2d6. | Butazolidin 200mg tablet |
| j2d8. | Butazone 200mg tablet |
| j2e.. | Piroxicam |
| j2e1. | Piroxicam 10mg capsule |
| j2e3. | Feldene 10mg capsule |
| j2ee. | Flamatrol 10mg capsule |
| j2e8. | Larapam 10mg capsule |
| j2eA. | Piroflam 10 capsule |
| j2ea. | Pirozip 10 capsule |
| j2e2. | Piroxicam 20mg capsule |
| j2e4. | Feldene 20mg capsule |
| j2ef. | Flamatrol 20mg capsule |
| j2e9. | Larapam 20mg capsule |
| j2eB. | Piroflam 20 capsule |
| j2eb. | Pirozip 20 capsule |
| j2ex. | Piroxicam 10mg dispersible tablet |
| j2e5. | Feldene 10mg dispersible tablet |
| j2ey. | Piroxicam 20mg dispersible tablet |
| j2e6. | Feldene 20mg dispersible tablet |
| j2ew. | Piroxicam 20mg melt tablet |
| j2ec. | Feldene Melt 20mg tablet |
| j2ev. | Piroxicam 20mg/1mL injection |
| j2ed. | Feldene IM 20mg/1mL injection |
| j2ez. | Piroxicam 20mg suppository |
| j2e7. | Feldene 20mg suppository |
| ja1y. | Piroxicam 0.5% gel |
| x00Ml | Feldene 0.5% gel |
| ja1M. | Feldene 0.5% topical gel 112g |
| ja1z. | Feldene 0.5% topical gel 60g |
| x03kk | Feldene P 0.5% gel |
| ja2O. | Feldene P 0.5% gel 30g |
| ja2P. | Feldene P 0.5% gel 7.5g |
| x026F | Feldene Sports 0.5% gel |
| ja1B. | Feldene sports gel 30g |
| j2i.. | Piroxicam-betadex |
| j2i1. | Piroxicam 20mg tablet |
| j2i2. | Brexidol 20mg tablet |
| j2f.. | Sulindac |
| j2fy. | Sulindac 100mg tablet |
| j2f1. | Clinoril 100mg tablet |
| j2fz. | Sulindac 200mg tablet |
| j2f2. | Clinoril 200mg tablet |
| j2l.. | Tenoxicam |
| j2l1. | Tenoxicam 20mg tablet |
| j2l2. | Mobiflex 20mg tablet |
| j2l5. | Tenoxicam 20mg effervescent tablet |
| j2l6. | Mobiflex 20mg effervescent tablet |
| j2l3. | Tenoxicam 20mg/sachet granules |
| j2l4. | Mobiflex Milk 20mg/sachet granules |
| j2l7. | Tenoxicam 20mg injection+diluent |
| j2l8. | Mobiflex vial 20mg injection+diluent |
| j2g.. | Tiaprofenic acid |
| j2gx. | Tiaprofenic acid 200mg tablet |
| j2g1. | Surgam 200mg tablet |
| j2gy. | Tiaprofenic acid 300mg tablet |
| j2g2. | Surgam 300mg tablet |
| j2g5. | Tiaprofenic acid 300mg m/r capsule |
| j2g4. | Surgam SA 300mg m/r capsule |
| j2gz. | Tiaprofenic acid 300mg/sachet granules |
| j2g3. | Surgam 300mg/sachet granules |
| j2h.. | Tolmetin |
| j2hy. | Tolmetin 200mg capsule |
| j2h2. | Tolectin 200mg capsule |
| j2hz. | Tolmetin 400mg capsule |
| j2h3. | Tolectin 400mg capsule |
| j2h1. | Tolectin DS 400mg capsules |

| **Group:** | IBUPROFEN_CD |
| --- | --- |
| **Description:** | Ibuprofen related codes |
|  |  |
| **READ CD** | **Description** |
| j28.. | IBUPROFEN [MUSCULOSKELETAL USE] |
| j281. | IBUPROFEN 200mg tablets |
| j282. | IBUPROFEN 400mg tablets |
| j283. | IBUPROFEN 600mg tablets |
| j284. | *APSIFEN 200mg tablets |
| j285. | *APSIFEN 400mg tablets |
| j286. | APSIFEN F 600mg effervescent tablets |
| j287. | BRUFEN 200mg tablets |
| j288. | BRUFEN 400mg tablets |
| j289. | BRUFEN 600mg tablets |
| j28A. | BRUFEN RETARD 800mg m/r tablets |
| j28B. | IBUPROFEN 800mg m/r tablets |
| j28C. | CUPROFEN 200mg tablets |
| j28D. | *ISISFEN 400mg tablets |
| j28E. | *LIDIFEN 200mg tablets |
| j28F. | *LIDIFEN 400mg tablets |
| j28G. | LIDIFEN 600mg tablets |
| j28H. | *CODAFEN CONTINUS m/r tablets |
| j28I. | NUROFEN PLUS tablets |
| j28J. | JUNIFEN 100mg/5mL sugar free suspension |
| j28K. | *IBUMED 400mg tablets |
| j28M. | *RIMAFEN 200mg tablets |
| j28N. | *RIMAFEN 400mg tablets |
| j28O. | IBUPROFEN+CODEINE PHOSPHATE 200mg/12.5mg tablets |
| j28P. | IBUPROFEN 600mg/sachet effervescent granules |
| j28R. | *INOVEN 200mg caplets |
| j28S. | RELCOFEN 200mg tablets |
| j28T. | *RELCOFEN 400mg tablets |
| j28U. | SOLPAFLEX tablets |
| j28V. | IBUPROFEN+CODEINE PHOSPHATE 200mg/12.8mg tablets |
| j28W. | *ADVIL 200mg tablets |
| j28X. | ADVIL EXTRA STRENGTH 400mg tablets |
| j28Y. | IBUPROFEN 100mg/5mL sugar free syrup |
| j28Z. | ANADIN IBUPROFEN 200mg tablets |
| j28a. | BRUFEN 100mg/5mL syrup |
| j28b. | EBUFAC 200mg tablets |
| j28c. | EBUFAC 400mg tablets |
| j28d. | *FENBID 300mg Spansules |
| j28e. | *IBULAR 200mg tablets |
| j28f. | *IBULAR 400mg tablets |
| j28g. | *IBUMETIN 200mg tablets |
| j28h. | *IBUMETIN 400mg tablets |
| j28i. | *IBUMETIN 600mg tablets |
| j28j. | *MOTRIN 200mg tablets |
| j28k. | *MOTRIN 400mg tablets |
| j28l. | *MOTRIN 600mg tablets |
| j28m. | *PAXOFEN 200mg tablets |
| j28n. | *PAXOFEN 400mg tablets |
| j28o. | *PAXOFEN 600mg tablets |
| j28p. | *MOTRIN 800mg tablets |
| j28q. | *ARTHROFEN 200mg tablets |
| j28r. | ARTHROFEN 400mg tablets |
| j28s. | JUNIFEN 100mg/5mL suspension 150mL |
| j28t. | *ARTHROFEN 600mg tablets |
| j28u. | *IBUPROFEN 800mg tablets |
| j28v. | BRUFEN 600mg granules |
| j28w. | IBUPROFEN 100mg/5mL syrup |
| j28x. | IBUPROFEN 200mg m/r capsules |
| j28y. | IBUPROFEN 300mg m/r capsules |
| j28z. | IBUPROFEN+CODEINE PHOSPHATE 300mg/20mg m/r tablets |
| j2p.. | IBUPROFEN [MUSCULOSKELETAL USE 2] |
| j2p1. | *NUROFEN ADVANCE 200mg tablets |
| j2p2. | NUROFEN FOR CHILDREN 100mg/5mL oral suspension |
| j2p3. | NUROFEN LONG LASTING 300mg m/r capsules |
| j2p4. | GALPROFEN 100mg/5mL oral suspension |
| j2p5. | LIBROFEM 200mg tablets |
| j2p6. | ORBIFEN FOR CHILDREN 100mg/5mL sugar free oral suspension |
| j2p7. | MANDAFEN 600mg tablets |
| j2p8. | FEVERFEN 100mg/5mL s/f oral suspension |
| j2p9. | NUROFEN FOR CHILDREN SINGLES 100mg/5mL sachets s/f oral suspension |
| j2pA. | IBUPROFEN 100mg/5mL sachets s/f oral suspension |
| j2pB. | MANDAFEN 400mg tablets |
| j2pC. | ORBIFEN 100mg/5mL sugar free oral suspension |
| j2pD. | NUROFEN MOBILE 200mg tablets |
| j2pE. | FENPAED 100mg/5mL sugar free oral suspension |
| j2pF. | MANDAFEN FOR CHILDREN 100mg/5mL sugar free oral suspension |
| j2pG. | CUPROFEN FOR CHILDREN 100mg/5mL sugar free oral suspension |
| j2pH. | NUROFEN 200mg tablets |
| j2pI. | NUROFEN 200mg caplets |
| j2pJ. | IBUPROFEN 200mg capsules |
| j2pK. | NUROFEN 200mg liquid capsules |
| j2pL. | CALPROFEN 100mg/5mL sugar free oral suspension |
| j2pM. | CUPROFEN PLUS tablets |
| j2pN. | ORBIFEN 100mg/5mL sachets sugar free oral suspension |
| j2pO. | CARE IBUPROFEN FOR CHILDREN 100mg/5mL s/f oral suspension |
| j2pP. | NUROFEN FOR CHILDREN strawberry 100mg/5mL s/f oral suspension |
| j2pQ. | GALPROFEN IBUPROFEN 200mg caplets |
| j2pR. | LLOYDS PHARMACY IBUPROFEN FOR CHILDREN 100mg/5mL s/f oral suspension |
| j2pS. | NUROFEN TENSION HEADACHE 200mg tablets |
| j2pT. | SOLPADEINE MIGRAINE IBUPROFEN & CODEINE tablets |
| j2pU. | NUROFEN MAXIMUM STRENGTH MIGRAINE PAIN 400mg Caplets |
| j2pV. | NUROFEN EXTRA STRENGTH 400mg liquid capsules |
| j2pW. | IBUPROFEN 400mg capsules |
| j2pX. | ANADIN ULTRA DOUBLE STRENGTH 400mg liquid capsules |
| j2pY. | GALPHARM MAXIMUM STRENGTH IBUPROFEN 400mg liquid capsules |
| j2pZ. | IBUCALM 200mg tablets |
| j2pa. | IBUCALM 400mg tablets |
| j2pb. | NUROFEN EXPRESS SOLUBLE 400mg/sachet oral powder |
| j2pc. | IBUPROFEN 400mg/sachet oral powder |

| **Group:** | PREDNISOLONE_CD |
| --- | --- |
| **Description:** | Prednisolone related codes |
|  |  |
| **READ CD** | **Description** |
| ai24. | Predsol 5mg suppository |
| ai27. | Prednisolone 5mg suppository |
| fe61. | Prednisolone 1mg tablet |
| fe62. | Prednisolone 5mg tablet |
| fe63. | Codelsol 32mg/2mL injection |
| fe64. | Delta-Phoricol 5mg tablet |
| fe65. | Deltacortril Enteric 2.5mg e/c tablet |
| fe66. | Deltacortril Enteric 5mg e/c tablet |
| fe67. | Deltalone 1mg tablet |
| fe68. | Deltalone 5mg tablet |
| fe69. | Deltastab 1mg tablet |
| fe6a. | Deltastab 5mg tablet |
| fe6b. | Deltastab 25mg/1mL injection |
| fe6c. | Precortisyl 1mg tablet |
| fe6d. | Precortisyl 5mg tablet |
| fe6e. | Precortisyl Forte 25mg tablet |
| fe6f. | Prednesol 5mg tablet |
| fe6g. | Sintisone 5mg tablet |
| fe6h. | Prednisolone 2.5mg e/c tablet |
| fe6i. | Prednisolone 5mg e/c tablet |
| fe6j. | Prednisolone 5mg soluble tablet |
| fe6k. | Prednisolone 50mg tablet |
| fe6l. | DILACORT 5mg gastro-resistant tablets |
| fe6m. | DILACORT 2.5mg gastro-resistant tablets |
| fe6u. | Prednisolone 32mg/2mL injection |
| fe6v. | Prednisolone 2.5mg tablet |
| fe6y. | Prednisolone 125mg/5mL injection |
| fe6z. | Prednisolone 25mg tablet |
| j44.. | Prednisolone acetate [musculoskeletal use] |
| j44z. | Prednisolone acetate 25mg/1mL injection |
| j45.. | Prednisolone sod phos [musc-sk] |
| j451. | Codelson 32mg/2mL injection |
| x00yP | Oral prednisolone |
| x01Nm | Parenteral prednisolone |
| x02M5 | Prednisolone product |

| **Group:** | VITAMIN_D |
| --- | --- |
| **Description:** | Vitamin D related codes |
|  |  |
| **READ CD** | **Description** |
| ip… | Vitamin D product |
| ip1.. | Alfacalcidol |
| ip16. | Alfacalcidol 250nanograms capsule |
| ip1d. | AlfaD 250nanograms capsule |
| ip11. | One-Alpha 250nanograms capsule |
| ip1z. | Alfacalcidol 500nanograms capsule |
| ip1h. | AlfaD 500nanograms capsule |
| ip1f. | One-Alpha 500nanograms capsule |
| ip17. | Alfacalcidol 1microgram capsule |
| ip1e. | AlfaD 1microgram capsule |
| ip12. | One-Alpha 1mcg capsule |
| ip18. | Alfacalcidol 200nanograms/mL oral solution |
| x00Xu | One-Alpha 200nanograms/mL oral solution |
| ip15. | One-Alpha 0.2micrograms/mL solution 60mL |
| ip1y. | Alfacalcidol 2micrograms/mL drops |
| ip1g. | One-Alpha 2micrograms/mL drops |
| ip19. | Alfacalcidol 1microgram/0.5mL injection |
| ip1b. | One-Alpha 1microgram/0.5mL injection |
| ip1a. | Alfacalcidol 2micrograms/1mL injection |
| ip1c. | One-Alpha 2micrograms/1mL injection |
| ip13. | One-Alpha 5micrograms/mL drops |
| ip14. | One-Alpha diluent |
| ip2.. | Calcitriol product |
| ip23. | Calcitriol 250nanograms capsule |
| ip21. | Rocaltrol 250nanograms capsule |
| ip24. | Calcitriol 500nanograms capsule |
| ip22. | Rocaltrol 500nanograms capsule |
| ip27. | Calcitriol 1microgram/1mL injection |
| ip25. | Calcijex 1microgram/1mL injection |
| ip28. | Calcitriol 2micrograms/1mL injection |
| ip26. | Calcijex 2micrograms/1mL injection |
| ip29. | Calcitriol 3micrograms/g ointment |
| x05dU | Silkis 3micrograms/g ointment |
| ip2A. | Silkis 3micrograms/g ointment 30g |
| ip2B. | Silkis 3micrograms/g ointment 100g |
| x05xq | Colecalciferol |
| x05xr | Alendronic acid + colecalciferol |
| fo4x. | Alendronic acid 70mg / colecalciferol 70micrograms tablet |
| fo45. | Fosavance tablet |
| ip3c. | Calcium carbonate+colecalciferol 1.25g/5micrograms tablet |
| ip3b. | Calcichew D3 tablet |
| ip3e. | Calcium carbonate+colecalciferol 1.25g/10micrograms tablet |
| ip39. | Calceos chewable tablet |
| ip3f. | Calcichew D3 Forte tablet |
| ip3g. | Calcium carbonate+colecalciferol 1.25g/440iu/sachet granules |
| ip3h. | Cacit D3 granules |
| ip3i. | Calcium carbonate+colecalciferol 1.5g/10micrograms chewable tablet |
| ip3j. | Adcal-D3 1.5g/10micrograms chewable tablet |
| ip3m. | ADCAL-D3 LEMON 1.5g/10micrograms chewable tablets |
| ip3n. | NATECAL D3 chewable tablets |
| ip3k. | Calcium phosphate+colecalciferol 3100mg/20micrograms/sachet powder for oral suspension |
| ip3l. | Calfovit D3 powder for oral suspension |
| ip4.. | Dihydrotachysterol |
| ip42. | Tachyrol 200micrograms tablet |
| ip43. | Dihydrotachysterol 250micrograms/mL oral solution |
| ip41. | AT 10 250micrograms/mL oral solution |
| x004E | Ergocalciferol product |
| ip33. | Calciferol 3000units/mL solution |
| ip31. | Calciferol 250micrograms tablet |
| x00ew | Calciferol 300micrograms tablet |
| ip3d. | Calciferol 1.25mg tablet |
| ip34. | Ergocalciferol 7.5mg(300,000units)/1mL injection |
| ip35. | Ergocalciferol 15mg(600,000units)/2mL injection |
| ip36. | Ergocalciferol 600,000units/1.5mL solution |
| ip38. | Sterogyl 15mg/1.5mL solution |
| x00ey | Calcium+ergocalciferol tablet |
| ip3a. | Calcium+ergocalciferol 400units tablet |
| ip32. | Calcium+vitamin D 500units tablet |
| ip37. | Chocovite 15micrograms tablet |
| ip3p. | CALCIUM CARBONATE+COLECALCIFEROL 1.5g/400iu effervescent tablets |
| ip3o. | ADCAL D3 DISSOLVE effervescent tablets |
| ip3.. | Cholecalciferol or ergocalciferol |
| ip3A. | COLECALCIFEROL 800iu tablets |
| ip3B. | DESUNIN 800iu tablets |
| ip3C. | SUNVIT-D3 1000iu tablets |
| ip3D. | SUNVIT-D3 10,000iu tablets |
| ip3E. | SUNVIT-D3 20,000iu tablets |
| ip3F. | SUNVIT-D3 50,000iu tablets |
| ip3G. | COLECALCIFEROL 1000iu tablets |
| ip3H. | COLECALCIFEROL 10,000iu tablets |
| ip3I. | COLECALCIFEROL 20,000iu tablets |
| ip3J. | COLECALCIFEROL 50,000iu tablets |
| ip3q. | SANDOCAL+D 600mg effervescent tablets |
| ip3r. | SANDOCAL+D 1200mg effervescent tablets |
| ip3s. | CALCICHEW D3 1.25g/400iu caplets |
| ip3t. | KALCIPOS-D 500mg/800iu chewable tablets |
| ip3u. | ADCAL D3 750mg/200iu caplets |
| ip3v. | CALCIUM CARBONATE+COLECALCIFEROL 750mg/200iu tablets |
| ip3w. | ACCRETE D3 tablets |
| ip3x. | CALCIUM CARBONATE+COLECALCIFEROL 1.5g/10micrograms tablets |
| ip3y. | FULTIUM-D3 800iu capsules |
| ip3z. | COLECALCIFEROL 800iu capsules |
| ip5.. | Paricalcitol |
| ip5z. | Paricalcitol 5micrograms/1mL solution for injection |
| ip51. | Zemplar 5micrograms/1mL solution for injection |
| ip5x. | PARICALCITOL 1microgram capsules |
| ip53. | ZEMPLAR 1microgram capsules |
| ip5w. | PARICALCITOL 2micrograms capsules |
| ip54. | ZEMPLAR 2micrograms capsules |
| ip5v. | PARICALCITOL 4micrograms capsules |
| ip55. | ZEMPLAR 4micrograms capsules |
| ip5y. | Paricalcitol 10micrograms/2mL solution for injection |
| ip52. | Zemplar 10micrograms/2mL solution for injection |

| **Group:** | LAB/PROCEDURES CODES |  |
| --- | --- | --- |
| **Description:** | Codes related to laboratory/procedures that showed a high correlation | |
|  |  |  |
| **READ CD** | **Description** | **Subgroup** |
| 44J3 | Serum creatinine | LAB_GR_1 |
| 44I5 | Serum sodium | LAB_GR_1 |
| 44I4 | Serum potassium | LAB_GR_1 |
| 42L.. | Basophil count | LAB_GR_2 |
| 42M.. | Lymphocyte count | LAB_GR_2 |
| 42K.. | Eosinophil count | LAB_GR_2 |
| 42J.. | Neutrophil count | LAB_GR_2 |
| 42N.. | Monocyte count | LAB_GR_2 |
| 42P.. | Platelet count | LAB_GR_3 |
| 42A.. | Mean corpuscular volume (MCV) | LAB_GR_3 |
| 426.. | Red blood cell (RBC) count | LAB_GR_3 |
| 428.. | Mean corpuscular haemoglobin (MCH) | LAB_GR_3 |

# Thomas et al’s Method

| **Group** RA codes | | |
| --- | --- | --- |
| **Description** Rheumatoid arthritis diagnosis codes | | |
|  | | |
| **READ CD** | **Description** | INTENSITY |
| N040.00 | Rheumatoid arthritis | 2 |
| N042200 | Rheumatoid nodule | 3 |
| 14G1.00 | H/O: rheumatoid arthritis | 2 |
| N040P00 | Seronegative rheumatoid arthritis | 4 |
| N040T00 | Flare of rheumatoid arthritis | 2 |
| N047.00 | Seropositive errosive rheumatoid arthritis | 1 |
| H570.00 | Rheumatoid lung | 3 |
| N04X.00 | Seropositive rheumatoid arthritis, unspecified | 1 |
| 66H..13 | Rheumatoid arthrit. monitoring | 2 |
| N040200 | Rheumatoid arthritis of shoulder | 2 |
| N041.00 | Felty's syndrome | 3 |
| N005.00 | Adult Still's Disease | 2 |
| N04..00 | Rheumatoid arthritis and other inflammatory polyarthropathy | 4 |
| N04y012 | Fibrosing alveolitis associated with rheumatoid arthritis | 3 |
| N040N00 | Rheumatoid vasculitis | 3 |
| N040S00 | Rheumatoid arthritis - multiple joint | 2 |
| N04y000 | Rheumatoid lung | 3 |
| N04y200 | Adult-onset Still's disease | 2 |
| N043300 | Monarticular juvenile rheumatoid arthritis |  |
| N042z00 | Rheumatoid arthropathy + visceral/systemic involvement NOS | 3 |
| N040900 | Rheumatoid arthritis of PIP joint of finger | 2 |
| N040800 | Rheumatoid arthritis of MCP joint | 2 |
| G5yA.00 | Rheumatoid carditis | 3 |
| N040100 | Other rheumatoid arthritis of spine | 2 |
| N040000 | Rheumatoid arthritis of cervical spine | 2 |
| N042100 | Rheumatoid lung disease | 3 |
| N043100 | Acute polyarticular juvenile rheumatoid arthritis |  |
| N040700 | Rheumatoid arthritis of wrist | 2 |
| N040B00 | Rheumatoid arthritis of hip | 2 |
| N042.00 | Other rheumatoid arthropathy + visceral/systemic involvement | 3 |
| G5y8.00 | Rheumatoid myocarditis | 3 |
| N040D00 | Rheumatoid arthritis of knee | 2 |
| N040K00 | Rheumatoid arthritis of 1st MTP joint | 2 |
| N040F00 | Rheumatoid arthritis of ankle | 2 |
| N040R00 | Rheumatoid nodule | 3 |
| Nyu1G00 | [X]Seropositive rheumatoid arthritis, unspecified | 1 |
| N040500 | Rheumatoid arthritis of elbow | 2 |
| F371200 | Polyneuropathy in rheumatoid arthritis | 3 |
| N040A00 | Rheumatoid arthritis of DIP joint of finger | 2 |
| N040600 | Rheumatoid arthritis of distal radio-ulnar joint | 2 |
| Nyu1200 | [X]Other specified rheumatoid arthritis | 2 |
| N040H00 | Rheumatoid arthritis of talonavicular joint | 2 |
| N040J00 | Rheumatoid arthritis of other tarsal joint | 2 |
| N040G00 | Rheumatoid arthritis of subtalar joint | 2 |
| Nyu1100 | [X]Other seropositive rheumatoid arthritis | 2 |
| N040L00 | Rheumatoid arthritis of lesser MTP joint | 2 |
| 38DZ.00 | Disease activity score in rheumatoid arthritis | 2 |
| N040C00 | Rheumatoid arthritis of sacro-iliac joint | 2 |
| N040400 | Rheumatoid arthritis of acromioclavicular joint | 2 |
| 7P20300 | Delivery of rehabilitation for rheumatoid arthritis | 4 |

| **Group** Alt.arthropathy | |
| --- | --- |
| **Description** Alternative arthropathy codes | |
|  | |
| **READ CD** | **Description** |
| M160.00 | Psoriatic arthropathy |
| C34..00 | Gout |
| N100.00 | Ankylosing spondylitis |
| A993.11 | Reiter's syndrome |
| N02..00 | Crystal arthropathies |
| N023.00 | Gouty arthritis |
| N01w.00 | Reactive arthropathy, unspecified |
| N02..14 | Pseudogout |
| N02..11 | Chondrocalcinosis |
| N013.00 | Postdysenteric reactive arthropathy |
| A993.00 | Reiter's disease / syndrome |
| C340.00 | Gouty arthropathy |
| C342.00 | Idiopathic gout |
| M160z00 | Psoriatic arthropathy NOS |
| N023z00 | Gouty arthritis NOS |
| N015.00 | Arthropathy associated with other viral diseases |
| A56x000 | Arthritis due to rubella |
| N02..13 | Crystal synovitis |
| E245.11 | LSD dependence |
| A985000 | Gonococcal arthritis |
| C34z.00 | Gout NOS |
| A15..12 | Tuberculous arthritis |
| 6693 | Joints gout affected |
| A022300 | Salmonella arthritis |
| N02..12 | Crystal arthritis |
| N011x00 | Sexually acquired reactive arthropathy of multiple sites |
| M160100 | Distal interphalangeal psoriatic arthropathy |
| N01w900 | Reactive arthropathy of hip |
| N10..00 | Inflammatory spondylopathies |
| N023700 | Gouty arthritis of the ankle and foot |
| N014.00 | Arthropathy associated with other bacterial diseases |
| N015z00 | Arthropathy associated with other viral disease NOS |
| N021300 | Chondrocalcinosis-pyrophosphate crystals, of the forearm |
| N10yz00 | Other inflammatory spondylopathies NOS |
| N02zz00 | Crystal arthropathy NOS |
| N010A00 | Arthritis in Lyme disease |
| N10y.00 | Other inflammatory spondylopathies |
| C344.00 | Drug-induced gout |
| N02zD00 | Crystal arthropathy NOS, of wrist |
| N023300 | Gouty arthritis of the forearm |
| N017.11 | Arthropathy due to parasitic infection |
| N02y800 | Hydroxyapatite deposition disease |
| N016.11 | Arthropathy due to fungal infection |
| N023600 | Gouty arthritis of the lower leg |
| N011.12 | Arthropathy in Reiter's disease |
| N016700 | Arthropathy associated with mycoses, of the ankle and foot |
| N01wD00 | Reactive arthropathy of ankle |
| N022.00 | Chondrocalcinosis, unspecified |
| N015x00 | Arthropathy with other viral disease, of multiple sites |
| N023400 | Gouty arthritis of the hand |
| N01w000 | Reactive arthropathy of shoulder |
| N01wB00 | Reactive arthropathy of knee |
| N016300 | Arthropathy associated with mycoses, of the forearm |
| Nyu0300 | [X]Other reactive arthropathies |
| N02zK00 | Crystal arthropathy NOS, of knee |
| N021611 | Chondrocalcinosis due to pyrophosphate crystals of the knee |
| N02z.00 | Crystal arthropathy NOS |
| N014800 | Arthropathy in Whipple's disease |
| N023x00 | Gouty arthritis of multiple sites |
| N01w500 | Reactive arthropathy of wrist |
| N10y000 | Inflammatory spondylopathies in diseases EC |
| N012011 | Behcet's syndrome arthropathy |
| Nyu1300 | [X]Other psoriatic arthropathies |
| N023y00 | Gouty arthritis of other specified site |
| F464200 | Myotonic cataract |
| N01w300 | Reactive arthropathy of elbow |
| N015500 | Arthropathy with other viral disease, of pelvic region/thigh |
| N02y100 | Other crystal arthropathies of the shoulder |
| N012x00 | Arthropathy in Behcet's syndrome of multiple sites |
| N01wK00 | Reactive arthropathy of IP joint of toe |
| N011.00 | Sexually acquired reactive arthropathy |
| N020700 | Chondrocalcinosis-dicalcium phosphate, of the ankle and foot |
| N022y00 | Chondrocalcinosis unspecified, of other specified site |
| N021100 | Chondrocalcinosis-pyrophosphate crystals, of shoulder region |
| N02z300 | Crystal arthropathy NOS, of the forearm |
| N021600 | Chondrocalcinosis-pyrophosphate crystals, of the lower leg |
| N02z600 | Crystal arthropathy NOS, of the lower leg |
| N020.00 | Chondrocalcinosis due to dicalcium phosphate crystals |
| N02zL00 | Crystal arthropathy NOS, of tibio-fibular joint |
| N015600 | Arthropathy with other viral disease, of lower leg |
| N021y00 | Chondrocalcinosis-pyrophosphate crystals, of other spec site |
| N015400 | Arthropathy with other viral disease, of hand |
| N021700 | Chondrocalcinosis-pyrophosphate crystals, of ankle and foot |
| N016.00 | Arthropathy associated with mycoses |
| N013700 | Postdysenteric reactive arthropathy of the ankle and foot |
| N015700 | Arthropathy with other viral disease, of ankle and foot |
| N012.00 | Arthropathy in Behcet's syndrome |
| N015300 | Arthropathy with other viral disease, of forearm |
| N01w600 | Reactive arthropathy of MCP joint |
| N020600 | Chondrocalcinosis-dicalcium phosphate, of the lower leg |
| N02z400 | Crystal arthropathy NOS, of the hand |
| N022600 | Chondrocalcinosis unspecified, of the lower leg |
| N015000 | Arthropathy with other viral disease, of unspecified site |
| N017100 | Helminthiasis with arthropathy of the shoulder region |
| N022000 | Chondrocalcinosis unspecified, of unspecified site |
| N02y.00 | Other crystal arthropathies |
| N02yx00 | Other crystal arthropathies of multiple sites |
| N021x00 | Chondrocalcinosis-pyrophosphate crystals, of multiple sites |
| N021400 | Chondrocalcinosis-pyrophosphate crystals, of the hand |
| N011z00 | Sexually acquired reactive arthropathy NOS |
| N014700 | Arthropathy with other bacterial disease, of ankle and foot |
| N021000 | Chondrocalcinosis-pyrophosphate crystals, of unspec site |
| N012000 | Arthropathy in Behcet's syndrome of unspecified site |
| N023100 | Gouty arthritis of the shoulder region |
| N014000 | Arthropathy with other bacterial disease, of unspec site |
| N011600 | Sexually acquired reactive arthropathy of the lower leg |
| N016z00 | Arthropathy associated with mycoses NOS |
| N022500 | Chondrocalcinosis unspecified, of the pelvic region/thigh |
| N021z00 | Chondrocalcinosis due to pyrophosphate crystals, NOS |
| N02z700 | Crystal arthropathy NOS, of the ankle and foot |
| N023800 | Gouty arthritis of toe |
| N016400 | Arthropathy associated with mycoses, of the hand |
| N02y600 | Other crystal arthropathies of the lower leg |
| N02zF00 | Crystal arthropathy NOS, of PIP joint of finger |
| N020400 | Chondrocalcinosis-dicalcium phosphate, of the hand |
| N01wA00 | Reactive arthropathy of sacro-iliac joint |
| N02yz00 | Other crystal arthropathy NOS |

| **Group** DMARDs product |
| --- |
| **Description** DMARDs product codes |

| **Group** DMARDs product | |
| --- | --- |
| **Description** DMARDs product codes | |
|  | |
| **READ_CD** | **Description** |
|  | Chloroquine sulphate 68mg/5ml oral solution |
| j521. | Penicillamine 50mg tablets |
| h71z. | Azathioprine 50mg powder for solution for injection vials |
| j513. | Myocrisin 10mg/0.5ml solution for injection ampoules (Sanofi) |
| aa6z. | Sulfasalazine 3g/100ml enema |
| aa64. | Salazopyrin 3g/100ml Enema (Pharmacia Ltd) |
| aa62. | Salazopyrin EN-Tabs 500mg (Pfizer Ltd) |
| j551. | Salazopyrin EN-Tabs 500mg (Pfizer Ltd) |
| ej26. | Chloroquine phosphate 250mg tablets |
| h71y. | Azathioprine 25mg tablets |
|  | Chloroquine sulphate 200mg tablets |
| ej25. | Nivaquine 272.5mg(200mg base)/5ml Injection (Aventis Pharma) |
| aa6v. | Sulfasalazine 500mg gastro-resistant tablets |
| ejC.. | Chloroquine phosphate 250mg tablets and Proguanil 100mg tablets |
| h71x. | Azathioprine 50mg tablets |
| j523. | Penicillamine 250mg tablets |
| j522. | Penicillamine 125mg tablets |
| h712. | Imuran 25mg Tablet (Wellcome Medical Division) |
| j54z. | Hydroxychloroquine 200mg tablets |
|  | Azathioprine capsules |
| h341. | Methotrexate 2.5mg tablets |
| h342. | Methotrexate 10mg tablets |
| h82A. | Neoral 25mg capsules (Novartis Pharmaceuticals UK Ltd) |
| h82C. | Neoral 100mg capsules (Novartis Pharmaceuticals UK Ltd) |
| aa61. | Salazopyrin 500mg Tablet (Pharmacia Ltd) |
| h82x. | Ciclosporin 100mg/ml oral solution sugar free |
| h713. | Imuran 50mg Tablet (Wellcome Medical Division) |
| h82D. | Neoral 100mg/ml oral solution (Novartis Pharmaceuticals UK Ltd) |
| h829. | Ciclosporin 50mg capsules |
| h826. | Ciclosporin 25mg capsules |
| aa6y. | Sulfasalazine 500mg tablet |
| ej24. | Nivaquine 68mg/5ml Oral solution (Aventis Pharma) |
| ej23. | Nivaquine 200mg Tablet (Aventis Pharma) |
| j515. | Myocrisin 50mg/0.5ml solution for injection ampoules (Sanofi) |
| ej21. | Avloclor 250mg tablets (AstraZeneca UK Ltd) |
| j525. | Distamine 125mg tablets (Alliance Pharmaceuticals Ltd) |
| j514. | Myocrisin 20mg/0.5ml solution for injection ampoules (Sanofi) |
| j55z. | Sulfasalazine 500mg suppositories |
| h827. | Ciclosporin 100mg capsules |
| h824. | Sandimmun 25mg capsules (Novartis Pharmaceuticals UK Ltd) |
| j561. | Auranofin 3mg tablets |
| h82B. | Neoral 50mg capsules (Novartis Pharmaceuticals UK Ltd) |
| aa63. | Salazopyrin 500mg Suppository (Pharmacia Ltd) |
| j51z. | Sodium aurothiomalate 50mg/0.5ml solution for injection ampoules |
| ej31. | Plaquenil 200mg tablets (Sanofi) |
| j541. | Plaquenil 200mg tablets (Sanofi) |
| j59z. | Leflunomide 100mg tablets |
| j59x. | Leflunomide 10mg tablets |
| aa61. | Salazopyrin 500mg tablets (Pfizer Ltd) |
| aa6v. | Sulfasalazine 500mg tablets |
| ejC1. | Proguanil 100mg & chloroquine 250mg tablets |
| h871. | Adalimumab 40mg injection |
| h873. | Adalimumab 40mg injection |
| j59y. | Leflunomide 20mg tablets |
| ejC2. | Paludrine/Avloclor tablets anti-malarial travel pack (AstraZeneca UK Ltd) |
| h346. | Methotrexate 12.5mg/0.5ml solution for injection pre-filled syringes |
| h34z. | Methotrexate 10mg/0.4ml solution for injection pre-filled syringes |
| aa6u. | Sulfasalazine 250mg/5ml oral solution |
|  | Methotrexate 50mg/3ml Injection |
| h345. | Methotrexate 25mg/ml Injection |
| j526. | Distamine 250mg tablets (Alliance Pharmaceuticals Ltd) |
| h344. | Methotrexate 5mg/2ml solution for injection vials |
| mb56. | Oilatum scalp treatment shampoo (GlaxoSmithKline UK Ltd) |
| mb57. | Oilatum scalp treatment shampoo (GlaxoSmithKline UK Ltd) |
| aa63. | Salazopyrin 500mg suppositories (Pfizer Ltd) |
| ej2w. | Chloroquine phosphate 80mg/5ml oral solution |
| j51x. | Sodium aurothiomalate 10mg/0.5ml solution for injection ampoules |
| aa6u. | Sulfasalazine 250mg/5ml oral suspension |
| j524. | Distamine 50mg Tablet (Alliance Pharmaceuticals Ltd) |
| h711. | Azamune 50mg Tablet (Penn Pharmaceuticals Ltd) |
|  | Methotrexate 100mg/ml Injection |
| ej23. | Nivaquine 200mg tablets (Sanofi) |
| h718. | Azathioprine 10mg tablets |
| aa64. | Salazopyrin 3g/100ml enema (Pfizer Ltd) |
| h34p. | Maxtrex 2.5mg tablets (Pfizer Ltd) |
| j562. | Ridaura Tiltab 3mg tablets (Astellas Pharma Ltd) |
| h821. | Sandimmun 100mg/ml oral solution (Novartis Pharmaceuticals UK Ltd) |
| h825. | Sandimmun 100mg capsules (Novartis Pharmaceuticals UK Ltd) |
| aa65. | Salazopyrin 250mg/5ml oral suspension (Pfizer Ltd) |
| h345. | Methotrexate 20mg/0.8ml solution for injection pre-filled syringes |
| h34G. | Metoject 20mg/2ml solution for injection pre-filled syringes (medac UK) |
| h714. | Imuran 50mg powder for solution for injection vials (Aspen Pharma Trading Ltd) |
|  | Methotrexate sodium 25mg/ml Injection |
| ej24. | Nivaquine 68mg/5ml syrup (Sanofi) |
| h89w. | Enbrel 25mg powder and solvent for solution for injection vials (Pfizer Ltd) |
|  | Chloroquine sulphate 200mg/5ml solution for injection ampoules |
| aa6v. | Sulfasalazine 500mg suppositories |
| h828. | Sandimmun 50mg capsules (Novartis Pharmaceuticals UK Ltd) |
| h892. | Etanercept 25mg powder and solvent for solution for injection vials |
| h82E. | Ciclosporin 10mg capsules |
| h82F. | Neoral 10mg capsules (Novartis Pharmaceuticals UK Ltd) |
| h345. | Methotrexate 25mg/1ml solution for injection pre-filled syringes |
| j591. | Arava 10mg tablets (Sanofi) |
|  | Methotrexate 15mg/0.6ml solution for injection pre-filled syringes |
|  | Methotrexate 7.5mg/0.3ml solution for injection pre-filled syringes |
| j51y. | Sodium aurothiomalate 20mg/0.5ml solution for injection ampoules |
| h8Bz. | Infliximab 100mg powder for solution for infusion vials |
|  | Methotrexate 2.5mg/5ml oral suspension |
| j592. | Arava 20mg tablets (Sanofi) |
|  | Methotrexate 22.5mg/0.9ml solution for injection pre-filled syringes |
| aa65. | Salazopyrin 250mg/5ml Liquid (Pharmacia Ltd) |
|  | Methotrexate sodium 2.5mg Tablet |
| j593. | Arava 100mg tablets (Sanofi) |
|  | Methotrexate 17.5mg/0.7ml solution for injection pre-filled syringes |
| h717. | Oprisine 50mg Tablet (Opus Pharmaceuticals Ltd) |
| h89z. | Enbrel 50mg powder and solvent for solution for injection vials (Wyeth Pharmaceuticals) |
|  | Ciclosporin 50mg/ml concentrate solution infusion |
| j528. | Pendramine 250mg Tablet (Viatris Pharmaceuticals Ltd) |
| aa66. | Sulfasalazine 500mg gastro-resistant tablets (Actavis UK Ltd) |
| h341. | Methotrexate 2.5mg tablets (Mercury Pharma Group Ltd) |
| h34q. | Maxtrex 10mg tablets (Pfizer Ltd) |
| h347. | METHOTREXATE 25MG/1ML |
| h348. | METHOTREXATE 25MG/1ML |
| h715. | Immunoprin 50mg tablets (Ashbourne Pharmaceuticals Ltd) |
| h8B1. | Remicade 100mg powder for solution for infusion vials (Merck Sharp & Dohme Ltd) |
|  | Azathioprine 50mg/5ml oral solution |
| j552. | Sulazine EC 500mg tablets (Genesis Pharmaceuticals Ltd) |
| ej22. | Malarivon 80mg/5ml syrup (Wallace Manufacturing Chemists Ltd) |
| h872. | Humira 40mg Injection (Abbott Laboratories Ltd) |
| h874. | Humira 40mg Injection (Abbott Laboratories Ltd) |
| h34C. | Methotrexate 25mg/2.5ml solution for injection pre-filled syringes |
| h349. | Methotrexate 50mg/2ml Injection |
| h34b. | Methotrexate 50mg/2ml Injection |
| h24B. | Methotrexate 20mg/2ml solution for injection pre-filled syringes |
| h716. | Berkaprine 50mg Tablet (Rorer Pharmaceuticals Ltd) |
| h891. | Etanercept 50mg powder and solvent for solution for injection vials |
| h822. | Sandimmun 50mg/ml Concentrate for solution for infusion (Novartis Pharmaceuticals UK Ltd) |
| h34r. | Maxtrex 2.5mg/ml Injection (Pharmacia Ltd) |
| h34D. | Metoject 15mg/1.5ml solution for injection pre-filled syringes (medac UK) |
| h34F. | Metoject 15mg/1.5ml solution for injection pre-filled syringes (medac UK) |
| h34A. | Methotrexate 15mg/1.5ml solution for injection pre-filled syringes |
| h34.. | METHOTREXATE |
| h3G.. | METHOTREXATE |
|  | Methotrexate 27.5mg/1.1ml solution for injection pre-filled syringes |
|  | Methotrexate 12.5mg/5ml oral suspension |
| hh11. | Rituximab 100mg/10ml solution for infusion vials |
| h34c. | Methotrexate 500mg/vial sterile powder |
| h71x. | Azathioprine 50mg tablets (IVAX Pharmaceuticals UK Ltd) |
|  | Hydroxychloroquine 200mg/5ml oral solution |
| j527. | Pendramine 125mg Tablet (Viatris Pharmaceuticals Ltd) |
| h719. | Imuran 10mg Tablet (Wellcome Medical Division) |
|  | Methotrexate 30mg/1.2ml solution for injection pre-filled syringes |
| h341. | Methotrexate 2.5mg Tablet (Pharmacia Ltd) |
| j523. | Penicillamine 250mg tablets (Actavis UK Ltd) |
| h346. | Methotrexate 5mg/0.2ml solution for injection pre-filled syringes |
| j522. | Penicillamine 125mg Tablet (IVAX Pharmaceuticals UK Ltd) |
| h71x. | Azathioprine 50mg tablets (Kent Pharmaceuticals Ltd) |
| j522. | Penicillamine 125mg tablets (Actavis UK Ltd) |
| j523. | Penicillamine 250mg tablets (A A H Pharmaceuticals Ltd) |
| aa6y. | Sulfasalazine 500mg tablets (A A H Pharmaceuticals Ltd) |
| aa6z. | Sulfasalazine 3g/100ml retention enema |
| aa66. | Sulfasalazine 500mg tablets (Actavis UK Ltd) |
| h71y. | Azathioprine 25mg tablets (A A H Pharmaceuticals Ltd) |
| h341. | Methotrexate 2.5mg tablets (Hospira UK Ltd) |
| h346. | Methotrexate 500mg/20ml solution for injection vials |
| h862. | Kineret 100mg/0.67ml solution for injection pre-filled syringes (Swedish Orphan Biovitrum Ltd) |
| h34z. | Methotrexate 10mg/1ml solution for injection pre-filled syringes |
| h34H. | Metoject 25mg/2.5ml solution for injection pre-filled syringes (medac UK) |
| j55z. | Sulfasalazine 500mg Gastro-resistant tablet (DDSA Pharmaceuticals Ltd) |
| aa66. | Sulfasalazine 500mg Tablet (Approved Prescription Services Ltd) |
| h345. | Methotrexate 20mg/0.8ml Injection (Central Homecare) |
| h71x. | Azathioprine 50mg tablets (Generics (UK) Ltd) |
| j55z. | Sulfasalazine 500mg tablets (Generics (UK) Ltd) |
| j523. | Penicillamine 250mg tablets (Generics (UK) Ltd) |
| h71x. | Azathioprine 50mg tablets (A A H Pharmaceuticals Ltd) |
| h71y. | Azathioprine 25mg tablets (Generics (UK) Ltd) |
| aa66. | Sulfasalazine 500mg Gastro-resistant tablet (Ceretron Ltd) |
| h342. | Methotrexate 10mg tablets (Hospira UK Ltd) |
| h893. | Etanercept 50mg injection solution |
| h895. | Etanercept 50mg injection solution |
| h34y. | Methotrexate 7.5mg/0.75ml solution for injection pre-filled syringes |
| h89v. | Enbrel 25mg/0.5ml solution for injection pre-filled syringes (Pfizer Ltd) |
|  | Azathioprine 50mg/5ml oral suspension |
|  | Methotrexate 7.5mg/5ml oral suspension |
| h34F. | Metoject 7.5mg/0.75ml solution for injection pre-filled syringes (medac UK) |
| h894. | Etanercept 25mg/0.5ml solution for injection pre-filled syringes |
| h34x. | Methotrexate 1g/10ml solution for injection vials |
| hh12. | Rituximab 500mg/50ml solution for infusion vials |
| h89u. | Enbrel 50mg Solution for injection (Pfizer Consumer Healthcare Ltd) |
| h89x. | Enbrel 50mg Solution for injection (Pfizer Consumer Healthcare Ltd) |
| h861. | Anakinra 100mg/0.67ml solution for injection pre-filled syringes |
|  | Azathioprine 250mg/5ml oral solution |
|  | Methotrexate 10mg/5ml oral solution |
|  | Methotrexate 10mg/5ml oral suspension |
| h34E. | Metoject 10mg/1ml solution for injection pre-filled syringes (medac UK) |
| h82y. | Ciclosporin 50mg/1ml solution for infusion ampoules |
| hh11. | Rituximab 10mg/ml concentrated intravenous infusion |
| h718. | Azathioprine 10mg capsules |
| j522. | Penicillamine 125mg tablets (Generics (UK) Ltd) |
| h34S. | Methotrexate 20mg/0.4ml solution for injection pre-filled syringes |
| h34L. | Metoject 7.5mg/0.15ml solution for injection pre-filled syringes (medac UK) |
| h34Q. | Methotrexate 15mg/0.3ml solution for injection pre-filled syringes |
| h34P. | Metoject 15mg/0.3ml solution for injection pre-filled syringes (medac UK) |
| h34V. | Metoject 15mg/0.3ml solution for injection pre-filled syringes (medac UK) |
| h34R. | Metoject 20mg/0.4ml solution for injection pre-filled syringes (medac UK) |
| h34T. | Metoject 25mg/0.5ml solution for injection pre-filled syringes (medac UK) |
| h34M. | Methotrexate 7.5mg/0.15ml solution for injection pre-filled syringes |
| h34U. | Methotrexate 25mg/0.5ml solution for injection pre-filled syringes |
| h34N. | Metoject 10mg/0.2ml solution for injection pre-filled syringes (medac UK) |
| h34O. | Methotrexate 10mg/0.2ml solution for injection pre-filled syringes |
| h89y. | Enbrel Paediatric 25mg powder and solvent for solution for injection vials (Pfizer Ltd) |
|  | Methotrexate 5g/50ml solution for infusion vials |
| h341. | Methotrexate 2.5mg tablets (Wockhardt UK Ltd) |
| h8Fz. | Tocilizumab 80mg/4ml solution for infusion vials |
|  | Methotrexate sodium 2.5mg Tablet (Wyeth Pharmaceuticals) |
| h71x. | Azathioprine 50mg tablets (Teva UK Ltd) |
| h71x. | Azathioprine 50mg Tablet (C P Pharmaceuticals Ltd) |
| j55z. | Sulfasalazine 500mg Tablet (Berk Pharmaceuticals Ltd) |
| h82I. | Deximune 50mg capsules (Dexcel-Pharma Ltd) |
| h82J. | Deximune 100mg capsules (Dexcel-Pharma Ltd) |
| h82H. | Deximune 25mg capsules (Dexcel-Pharma Ltd) |
| h82z. | Ciclosporin 250mg/5ml solution for infusion ampoules |
| h713. | Imuran 50mg tablets (Aspen Pharma Trading Ltd) |
| h712. | Imuran 25mg tablets (Aspen Pharma Trading Ltd) |
| h71x. | Azathioprine 50mg tablets (Actavis UK Ltd) |
| h8G2. | Cimzia 200mg/1ml solution for injection pre-filled syringes (UCB Pharma Ltd) |
| h8G1. | Certolizumab pegol 200mg/1ml solution for injection pre-filled syringes |
| aa6y. | Sulfasalazine 500mg gastro-resistant tablets (A A H Pharmaceuticals Ltd) |
|  | Chloroquine sulphate 200mg/5ml solution for injection ampoules (Sanofi) |
| h34W. | Methotrexate 30mg/0.6ml solution for injection pre-filled syringes |
| j542. | Quinoric 200mg tablets (Bristol Laboratories Ltd) |
| h343. | Methotrexate 20mg/1ml solution for injection pre-filled syringes |
| h3G5. | Methotrexate 25mg/1.25ml solution for injection pre-filled syringes |
| h3G6. | Methotrexate 30mg/1.5ml solution for injection pre-filled syringes |
| h3G7. | Metoject 12.5mg/0.25ml solution for injection pre-filled syringes (medac UK) |
| h3GC. | Methotrexate 22.5mg/0.45ml solution for injection pre-filled syringes |
| h3G8. | Methotrexate 12.5mg/0.25ml solution for injection pre-filled syringes |
| h3GA. | Methotrexate 17.5mg/0.35ml solution for injection pre-filled syringes |
| h3GB. | Metoject 22.5mg/0.45ml solution for injection pre-filled syringes (medac UK) |
| h3G9. | Metoject 17.5mg/0.35ml solution for injection pre-filled syringes (medac UK) |
| h8Fy. | Tocilizumab 200mg/10ml solution for infusion vials |
